# USP18 Antagonizes Pyroptosis by Facilitating Selective Autophagic Degradation of Gasdermin D

**DOI:** 10.34133/research.0380

**Published:** 2024-05-22

**Authors:** Liqiu Wang, Mengqiu Li, Guangyu Lian, Shuai Yang, Yaoxing Wu, Jun Cui

**Affiliations:** ^1^ MOE Key Laboratory of Gene Function and Regulation, Guangdong Province Key Laboratory of Pharmaceutical Functional Genes, State Key Laboratory of Biocontrol, School of Life Sciences of Sun Yat-sen University, Guangzhou, Guangdong, China.; ^2^ Department of Critical Care Medicine, The First Affiliated Hospital of Sun Yat-sen University, Guangzhou, Guangdong, China.

## Abstract

As a key executioner of pyroptosis, Gasdermin D (GSDMD) plays a crucial role in host defense and emerges as an essential therapeutic target in the treatment of inflammatory diseases. So far, the understanding of the mechanisms that regulate the protein level of GSDMD to prevent detrimental effects and maintain homeostasis is currently limited. Here, we unveil that ubiquitin-specific peptidase 18 (USP18) works as a negative regulator of pyroptosis by targeting GSDMD for degradation and preventing excessive innate immune responses. Mechanically, USP18 recruits E3 ubiquitin ligase mind bomb homolog 2 (MIB2) to catalyze ubiquitination on GSDMD at lysine (K) 168, which acts as a recognition signal for the selective autophagic degradation of GSDMD. We further confirm the alleviating effect of USP18 on LPS-triggered inflammation in vivo. Collectively, our study demonstrates the role of USP18 in regulating GSDMD-mediated pyroptosis and reveals a previously unknown mechanism by which GSDMD protein level is rigorously controlled by selective autophagy.

## Introduction

The innate immune system provides the first line of host defense against pathogens [1,2]. Upon sensing pathogen-associated molecular patterns (PAMPs) or danger-associated molecular patterns (DAMPs), pattern-recognition receptors (PRRs, e.g., NLRP3, NLRC4, and AIM2) recruit the adaptor ASC and effector pro-caspase-1 to form inflammasome, subsequently triggering caspase-1 activation, interlukin-1 beta (IL-1β) and IL-18 secretion, and pyroptosis [3,4]. Pyroptosis, characterized by cell swelling, membrane rupture, and pro-inflammatory factor release, is an inflammatory form of regulated cell death and plays pivotal roles in the host defense against invading pathogens [5–7]. Gasdermin D (GSDMD), a key executioner of pyroptosis, is activated through cleavage by activated caspase-1 or cytosolic lipopolysaccharide (LPS)-activated caspase-4/5 (murine caspase-11). Inflammatory caspases (caspase-1/4/5/11) cleave GSDMD at its inhibitory interdomain linker and liberates the N-terminal of GSDMD (GSDMD-NT) that binds to membrane lipids and forms pores on the membrane, resulting in pyroptosis and inflammatory cascades [8,9]. Accumulating evidence demonstrated that aberrant activation of GSDMD is associated with a diverse array of diseases, such as atherosclerosis [10], rheumatoid arthritis [11], gout [12], and sepsis [13]. Hence, precise regulation of GSDMD activation is essential to prevent detrimental consequences and uphold homeostasis.

Accumulating evidence indicates that GSDMD activation is tightly regulated by multiple post-translational modifications (PTMs) [9]. Protein ubiquitination, catalyzed by E3 ubiquitin ligases and counter-regulated by deubiquitinating enzymes, is a reversible PTM that plays critical roles in controlling protein activity or stability [14–16]. Recently, a few studies have been reported that GSDMD is modified by ubiquitination. E3 ubiquitin ligase SYVN1 was identified to promote GSDMD-mediated pyroptosis via enhancing the ubiquitination of GSDMD at K203 and K204 [17]. Another report showed that E3 ubiquitin ligase TRIM21 promoted ubiquitination and oligomerization of GSDMD, resulting in increased pyroptosis [18]. In addition, the environmental toxicant sodium arsenite (NaAsO_2_) reduced the K48- and K63-linked ubiquitination of GSDMD, thereby promoting GSDMD protein levels and pyroptosis by inhibiting its degradation through the ubiquitin–proteasome system and the autophagy–lysosome pathway [19]. However, the detailed mechanisms underlying ubiquitination that regulate the stability of GSDMD during inflammasome activation remain to be fully elucidated.

Among various deubiquitinases, ubiquitin-specific peptidase 18 (USP18) plays a critical role in orchestrating the regulation of innate immune responses and multiple biological processes [20], including inflammation [21], innate antiviral immunity [22–24], autophagy [25–28], cell development [29], and cancer cell pyroptosis [30]. However, its function in GSDMD-mediated pyroptosis in inflammasome activator-treated macrophages remains to be investigated. Here, we demonstrate that deubiquitinase USP18 is a negative regulator of pyroptosis via reducing the protein stability of GSDMD. Mechanistically, USP18 acts as a key scaffold protein to recruit E3 ubiquitin ligase mind bomb homolog 2 (MIB2) to GSDMD, which mediates ubiquitination on GSDMD at K168 in an enzyme activity-independent manner. The ubiquitination of GSDMD serves as a recognition signal for selective autophagic degradation. Accordingly, overexpression of USP18 decreases the GSDMD protein levels and LPS-triggered inflammation in vivo. Our findings demonstrate that USP18 negatively modulates GSDMD-mediated pyroptosis through selective autophagy to avoid excessive and potentially harmful inflammation.

## Results

### USP18 interacts with GSDMD and impairs GSDMD-mediated pyroptosis

**Fig. 1.**
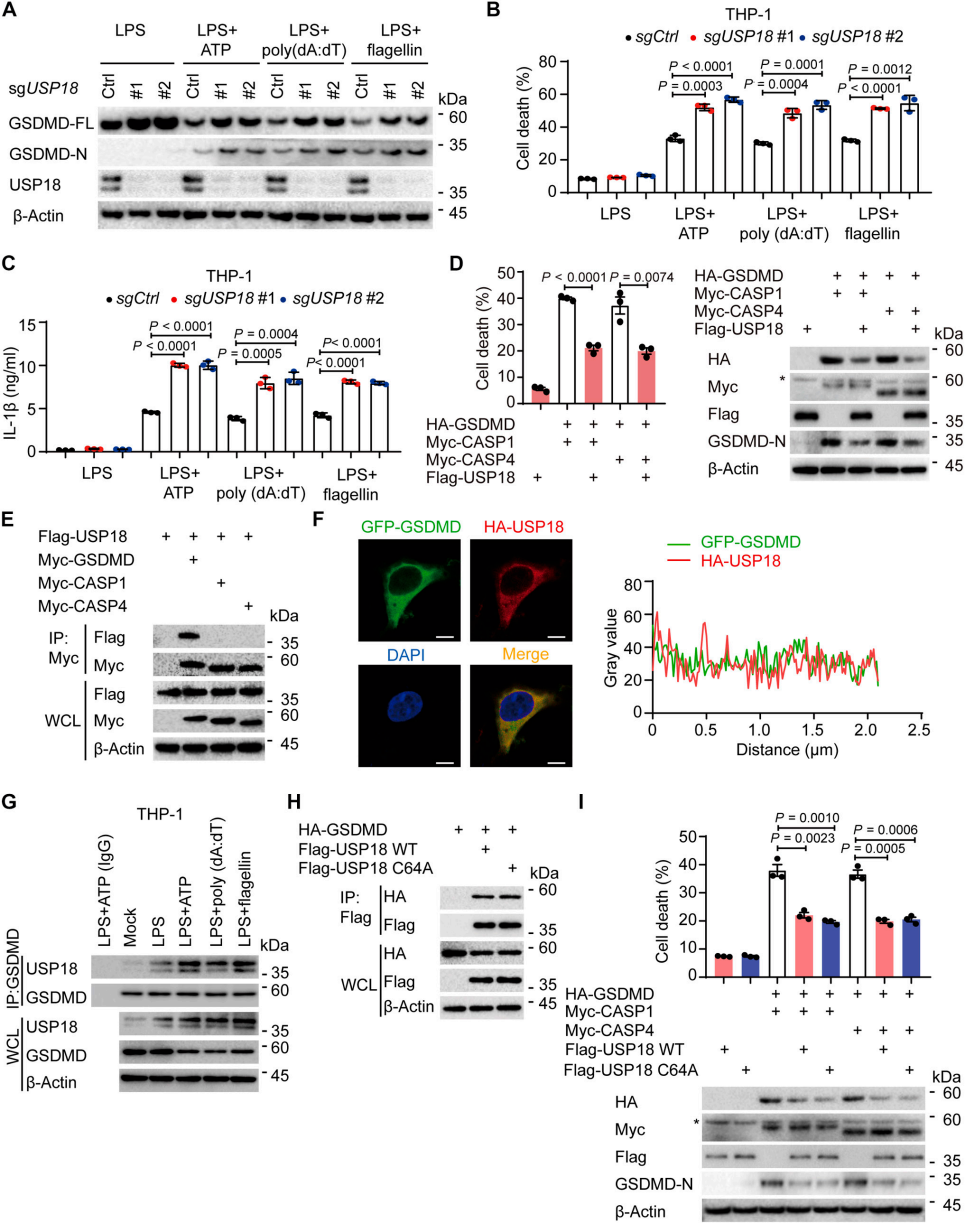
USP18 binds to GSDMD and inhibits GSDMD-mediated pyroptosis. (A to C) Wild type (WT, sg*Ctrl*) and *USP18*-knockout (KO, sg*USP18*#1/#2) THP-1-derived macrophages were primed with lipopolysaccharides (LPS, 200 ng/ml) for 3 h, followed by treatments of NLRP3 inflammasome agonist ATP (5 mM, 3 h), AIM2 inflammasome agonist poly (dA:dT) (2 μg/ml, 3 h), or NLRC4 inflammasome agonist flagellin (1 μg/ml, 3 h) treatment. Cell lysates were collected for immunoblot analysis (A). Cell death (B) and IL-1β production (C) were assessed by LDH (lactate dehydrogenase) release assay and ELISA analysis in the supernatants, respectively. (D) HEK293T cells were transfected with Flag-USP18, together with HA-empty vector (HA-EV), HA-GSDMD, Myc-caspase-1 (Myc-CASP1), or Myc-caspase-4 (Myc-CASP4), respectively. Cell lysates were collected for immunoblot analysis. Cell supernatants were collected for LDH release assay. (E) HEK293T cells were transfected with Flag-USP18, together with Myc-EV, Myc-GSDMD, Myc-CASP1, or Myc-CASP4, respectively. Cell lysates were collected for immunoprecipitation (IP) and immunoblot analysis. WCL, whole cell lysates. (F) HEK293T cells were transfected with GFP-GSDMD and HA-USP18. The colocalization between GSDMD (green) and USP18 (red) was examined by confocal microscopy. Scale bars, 10 μm. Gray value of indicated proteins were analyzed by ImageJ software. (G) THP-1-derived macrophages were primed with LPS (200 ng/ml) for 3 h, followed by ATP (5 mM, 3 h), poly (dA:dT) (2 μg/ml, 3 h), or flagellin (1 μg/ml, 3 h) treatment. Cell lysates were collected for immunoprecipitation and immunoblot analysis. (H) Immunoprecipitation and immunoblot analysis of HEK293T cells transfected with HA-GSDMD, together with Flag-EV, Flag-WT USP18, or its enzymatic activity mutant Flag-USP18 C64A. (I) Immunoblot analysis and LDH release assay of HEK293T cells transfected with HA-GSDMD and Myc-CASP1 or Myc-CASP4, together with Flag-EV, Flag-USP18 WT, or Flag-USP18 C64A. In (A), (D), (E), and (G) to (I), data are representative of 3 independent experiments with similar results. In (B) to (D) and (I), data are presented as mean values ± SEM, and *P* values were determined by unpaired 2-tailed Student’s *t* test of *n* = 3 independent biological experiments.

To investigate the potential functions of USP18 in GSDMD-mediated pyroptosis, we used effective and specific small interfering RNA (siRNA) targeting *USP18* to block *USP18* expression and found that the inflammasome activators (adenosine 5'-triphosphate [ATP], poly(dA:dT), or flagellin)-triggered cleavage of GSDMD (GSDMD-N levels) and cell death were enhanced in *USP18*-knockdown (KD) THP-1-derived macrophages (Fig. S1A and B). Previous studies indicated that USP18 inhibited inflammation by negatively regulating NF-κB signaling pathway [31,32], and GSDMD-N terminal fragments form transmembrane pores to enable the release of IL-1β [33–35]. Hence, combining previous studies with our observations provides a comprehensive explanation for the heightened IL-1β secretion mediated by inflammasome activation in *USP18*-KD THP-1-derived macrophages (Fig. S1C). To further validate the function of USP18, two *USP18*-knockout (KO) THP-1 cell lines were generated using the CRISPR/Cas9 technology. Consistently, the GSDMD cleavage, cell death, and IL-1β production induced by the inflammasome activators were increased in *USP18*-KO THP-1-derived macrophages (Fig. 1A to C). In addition, we further confirmed that USP18 overexpression could reduce CASP1/4-mediated GSDMD cleavage and GSDMD-mediated pyroptosis in HEK293T cells (Fig. 1D). Then, we observed that USP18 specifically interacted with GSDMD but not CASP1/4 (Fig. 1E). Reciprocal pull-down experiments also showed that GSDMD could associate with USP18 (Fig. S1D). Confocal analysis further confirmed that there was a strong colocalization between USP18 and GSDMD (Fig. 1F). GSDMD-USP18 association was increased by different inflammasome activators (Fig. 1G).

Given that USP18 is a deubiquitinase, we sought to investigate whether the enzymatic activity of USP18 is involved in the regulation of GSDMD-induced pyroptosis. We observed that, similarly to wild-type (WT) USP18, enzymatically inactive mutant USP18 C64A could interact with GSDMD (Fig. 1H). Moreover, the USP18 C64A mutant could still inhibit CASP1/4-mediated GSDMD cleavage and GSDMD-mediated pyroptosis in HEK293T cells (Fig. 1I). Together, these data suggest that USP18 binds to GSDMD and impairs GSDMD-mediated pyroptosis in an enzymatic activity-independent manner.

### USP18 enhances the autophagic degradation of GSDMD

**Fig. 2.**
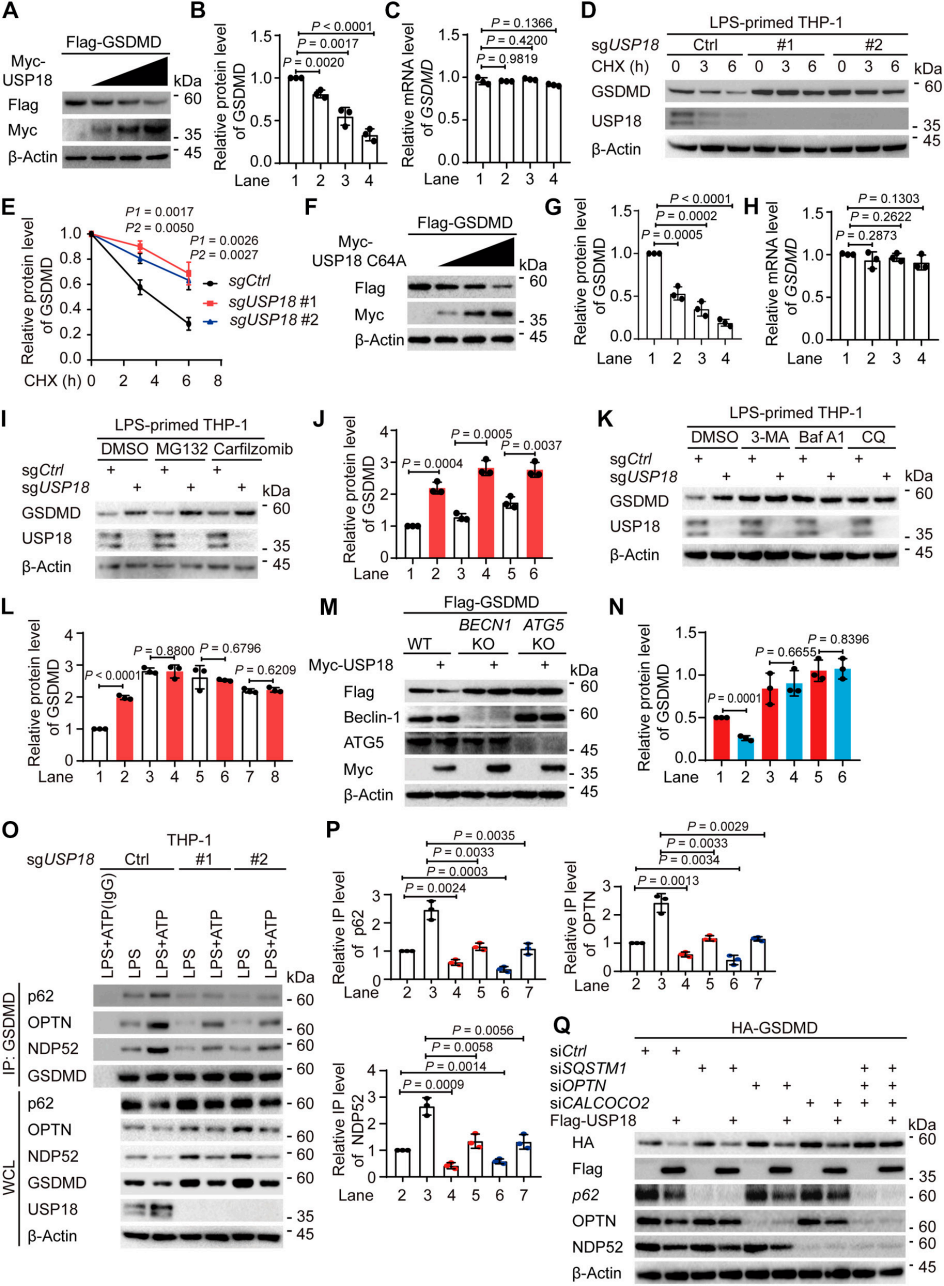
USP18 enhances the autophagic degradation of GSDMD. (A to C) HEK293T cells were transfected with Flag-GSDMD and increasing amounts of Myc-USP18. Cell lysates were collected for immunoblot analysis and quantitative real-time polymerase chain reaction (qPCR) analysis. Quantification of the relative protein level of GSDMD and the relative mRNA level of *GSDMD* was shown in (B) and (C), respectively. (D and E) Wild type (WT, sg*Ctrl*) and *USP18*-knockout (KO, sg*USP18*) THP-1-derived macrophages were primed with LPS (200 ng/ml) for 3 h, and followed by cycloheximide (CHX, 100 μg/ml) treatment at the indicated time points. Cell lysates were collected for immunoblot analysis (D). Quantitative analysis of the relative protein level of GSDMD was shown in (E). (F to H) Immunoblot analysis and qPCR analysis of HEK293T cells transfected with Flag-GSDMD and increasing amounts of Myc-USP18 C64A mutant. Quantification of the relative protein level of GSDMD and the relative mRNA level of *GSDMD* were shown in (G) and (H), respectively. (I to L) WT (sg*Ctrl*) and *USP18*-KO (sg*USP18*) THP-1-derived macrophages were pre-treated with LPS (200 ng/ml) for 3 h, then treated with DMSO (vehicle), MG132 (10 μM) or carfilzomib (100 nM), 3-methyladenine (3-MA, 10 mM), bafilomycin A1 (Baf A1, 0.2 μM), or chloroquine (CQ, 50 μM) for 6 h. Cell lysates were collected for immunoblot analysis (I and K), respectively. Quantitative analysis of the relative protein level of GSDMD was shown in (J) and (L), respectively. (M and N) Immunoblot analysis of WT, *BECN1*-KO, or *ATG5*-KO HEK293T cells transfected with Flag-GSDMD and Myc-USP18 (M). Quantitative analysis of the relative protein level of GSDMD was shown in (N). (O and P) WT (sg*Ctrl*) and *USP18*-KO (sg*USP18*) THP-1-derived macrophages were primed with LPS (200 ng/ml) for 3 h and followed by ATP (5 mM, 3 h) treatment. Cell lysates were collected for immunoprecipitation (IP) and immunoblot analysis. WCL, whole cell lysates (O). Quantification of the relative immunoprecipitated level of p62, OPTN, or NDP52 was shown in (P). (Q) HEK293T cells were transfected with control siRNA or *SQSTM1*/*p62*, *OPTN*, or (and) *CALCOCO2*/*NDP52* siRNA for 36 h, then the cells were transfected with HA-GSDMD and Flag-USP18 for 24 h. Cell lysates were collected for immunoblot analysis. In (A), (D), (F), (I), (K), (M), (O), and (Q), data are representative of 3 independent experiments with similar results. In (B), (E), (G), (J), (L), (N), and (P), quantification of the indicated protein levels was determined by Image Lab software, and data are presented as mean values ± SD; *P* values were determined by unpaired 2-tailed Student’s *t* test of *n* = 3 independent biological experiments. In (C) and (H), data are presented as mean values ± SEM, and *P* values were determined by unpaired 2-tailed Student’s *t* test of *n* = 3 independent biological experiments.

We next attempted to investigate the inhibitory function of USP18 in GSDMD-mediated pyroptosis through its interaction with GSDMD. In HEK293T cells, we observed that an increasing amount of USP18 substantially reduced the protein abundance of GSDMD, but not the mRNA level of *GSDMD* (Fig. 2A to C). To further confirm our findings, we knocked down or knocked out *USP18* in THP-1-derived macrophages and found that *USP18* deficiency resulted in increased GSDMD abundance (Fig. S2A and Fig. 2D). Cycloheximide (CHX) chase assay further showed that *USP18* deficiency also impaired the degradation rate of GSDMD (Fig. S2A and B and Fig. 2D and E). Additionally, we observed that the enzymatically inactive mutant of USP18 (C64A) could still promote GSDMD degradation without affecting its mRNA level (Fig. 2F to H), indicating that USP18 promoted the GSDMD degradation in an enzymatic activity-independent manner. Taken together, these results suggest that USP18 enhances the GSDMD degradation.

To investigate the degradation pathway through which USP18 targets GSDMD, we employed pharmacological inhibitors and observed that the degradation of GSDMD induced by USP18 was blocked by autophagy inhibitors 3-methyladenine (3-MA), bafilomycin A1 (Baf A1), ammonium chloride (NH_4_Cl), and chloroquine (CQ), but not the proteasome inhibitors MG132 or carfilzomib (Fig. S2C to F). Consistently, the treatment of autophagy inhibitors reversed the effects of *USP18* deficiency on GSDMD protein levels in THP-1-derived macrophages (Fig. S2G to J and Fig. 2I to L). These findings indicate that the degradation of GSDMD induced by USP18 predominantly occurs through the autolysosome pathway rather than the proteasome pathway. Subsequently, we observed a marked reduction in GSDMD protein levels upon initiation of autophagy through Earle’s balanced salt solution (EBSS) treatment. Moreover, USP18 was found to enhance the degradation of GSDMD induced by EBSS (Fig. S2K and L). In order to provide further evidence that GSDMD undergoes degradation via the autophagic pathway, we conducted experiments to assess the protein degradation of GSDMD in cells lacking *BECN1* or *ATG5*, both of which are crucial components for autophagy. We performed a CHX-chase assay and observed that the degradation of GSDMD was inhibited in *BECN1*- or *ATG5*-KO cells compared to WT cells (Fig. S2M to P). Additionally, we found that the degradation of GSDMD triggered by USP18 was almost completely abolished in *BECN1*- or *ATG5*-KO cells (Fig. 2M and N). These findings collectively support the notion that USP18 promotes the autophagic degradation of GSDMD.

Cargo receptors are essential components in the selective autophagic degradation process, responsible for delivering specific substrates to the autophagosome [36,37]. To identify the potential cargo receptors involved in the autophagic degradation of GSDMD, we examined the association between GSDMD and several previously reported cargo receptors, namely, SQSTM1/p62, OPTN, CALCOCO2/NDP52, NBR1, and TOLLIP [38,39]. We found that GSDMD could interact with p62, OPTN, NDP52, and NBR1 (Fig. S2Q), and *USP18* knockdown impaired the interactions between GSDMD and p62, OPTN, or NDP52, respectively, in HEK293T cells (Fig. S2Q and R). Consistent with the observation in *USP18*-KD HEK293T cells, the endogenous GSDMD-p62/OPTN/NDP52 association was restricted in *USP18*-KD/KO THP-1-derived macrophages with LPS alone or LPS plus ATP treatment (Fig. S2S and T and Fig. 2O and P). Additionally, single knockdown of *SQSTM1*/*p62*, *OPTN*, or *CALCOCO2*/*NDP52* could not completely block USP18-mediated GSDMD degradation, while USP18 failed to increase the degradation of GSDMD in triple knockdown cells (Fig. 2Q and Fig. S2U). Taken together, these findings strongly indicate that USP18 enhances the recognition of GSDMD by multiple cargo receptors, thereby promoting its subsequent autophagic degradation.

### USP18 increases the ubiquitination of GSDMD at K168

**Fig. 3.**
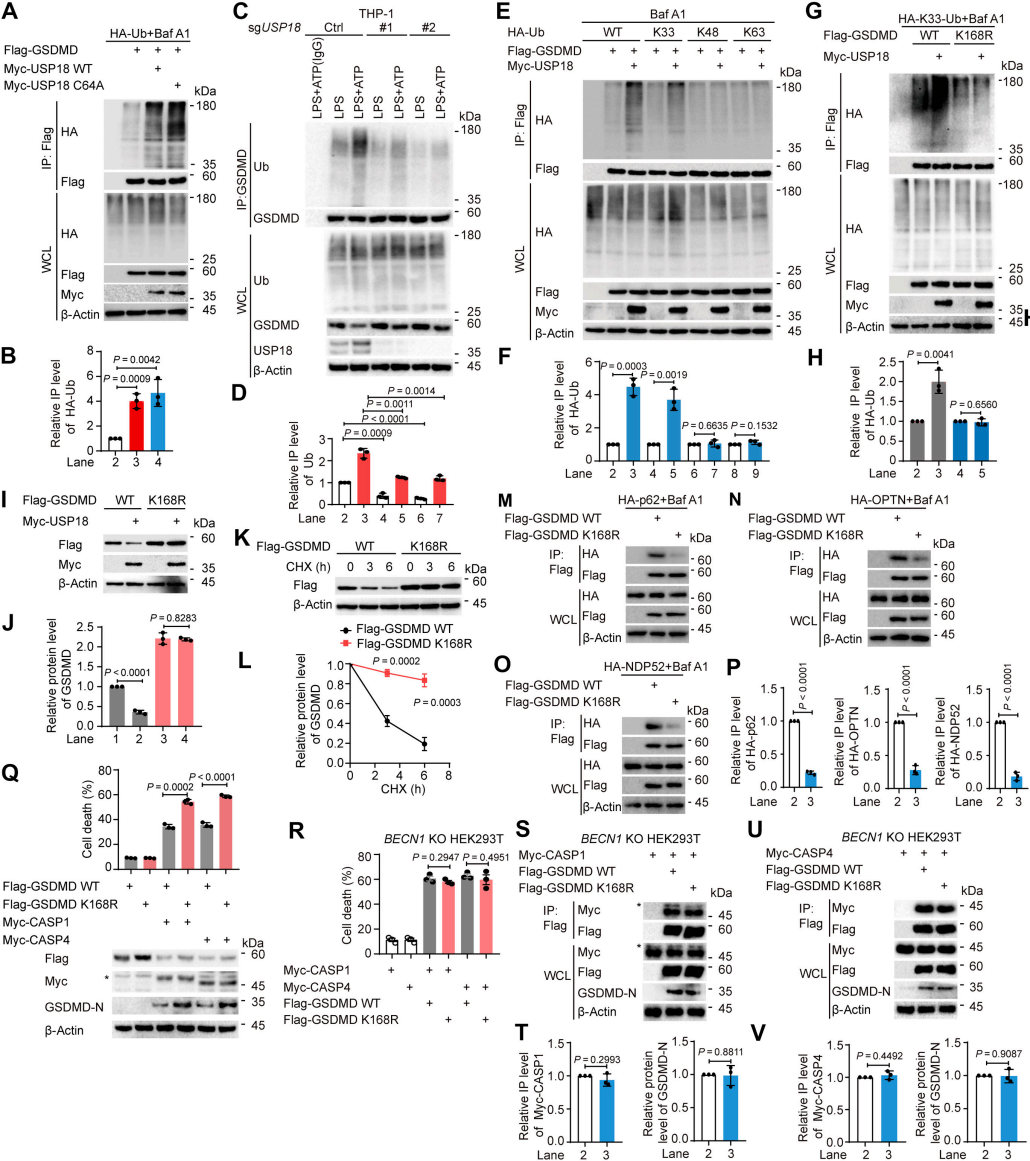
USP18 promotes the ubiquitination of GSDMD at K168 and its subsequent degradation. (A and B) HEK293T cells were transfected with HA-ubiquitin (HA-Ub) and Flag-GSDMD, together with wild type (WT) Myc-USP18 or Myc-USP18 C64A for 18 h, then treated with Baf A1 (0.2 μM) for 6 h. Cell lysates were collected for immunoprecipitation (IP) and immunoblot analysis. WCL, whole-cell lysates (A). Quantitative analysis of the relative immunoprecipitated level of HA-Ub was shown in (B). (C and D) WT (sg*Ctrl*) and *USP18*-KO (sg*USP18*) THP-1-derived macrophages were primed with LPS (200 ng/ml) for 3 h and followed by ATP (5 mM, 3 h) treatment. Cell lysates were collected for immunoprecipitation and immunoblot analysis (C). Quantification of the relative immunoprecipitated level of Ub was shown in (D). (E and F) HEK293T cells were transfected with Flag-GSDMD and HA-WT-Ub, HA-K33-Ub, HA-K48-Ub, or HA-K63-Ub, together with Myc-empty vector (EV) or Myc-USP18 for 18 h, then treated with Baf A1 (0.2 μM) for 6 h. Cell lysates were collected for immunoprecipitation and immunoblot analysis (E). Quantification of the relative immunoprecipitated level of HA-Ub was shown in (F). (G and H) HEK293T cells were transfected with HA-K33-Ub and Flag-GSDMD WT or Flag-GSDMD K168R, together with Myc-EV or Myc-USP18 for 18 h, then treated with Baf A1 (0.2 μM) for 6 h. Cell lysates were collected for immunoprecipitation and immunoblot analysis (G). Quantification of the relative immunoprecipitated level of HA-Ub was shown in (H). (I and J) Immunoblot analysis of HEK293T cells transfected with Flag-GSDMD or Flag-GSDMD K168R mutant, together with Myc-EV or Myc-USP18 (I). Quantitative analysis of the relative protein level of GSDMD was shown in (J). (K and L) HEK293T cells were transfected with Flag-GSDMD WT or Flag-GSDMD K168R mutant for 24 h, then treated with CHX (100 μg/ml) as indicated time points. Cell lysates were collected for immunoblot analysis (K). Quantitative analysis of the relative protein level of GSDMD was shown in (L). (M to P) HEK293T cells were transfected with HA-p62 (M), HA-OPTN (N), or HA-NDP52 (O), together with Flag-GSDMD WT or Flag-GSDMD K168R for 18 h, then treated with Baf A1 (0.2 μM) for 6 h. Cell lysates were collected for immunoprecipitation and immunoblot analysis (M to O). Quantitative analysis of the relative immunoprecipitated level of HA-p62, HA-OPTN, or HA-NDP52 was shown in (P). (Q) Immunoblot analysis and LDH release assay of HEK293T cells transfected with Flag-GSDMD WT or Flag-GSDMD K168R, together with Myc-CASP1 or Myc-CASP4. (R to V) *BECN1*-KO HEK293T cells were transfected with Myc-CASP1 or Myc-CASP4, together with Flag-GSDMD WT or Flag-GSDMD K168R for 24 h. Cell death was assessed by LDH (lactate dehydrogenase) release assay of cell supernatants (R). Cell lysates were collected for immunoprecipitation and immunoblot analysis (S and U). Quantitative analysis of the relative immunoprecipitated level of Myc-CASP1 or Myc-CASP4, or the relative protein level of GSDMD-N levels was shown in (T) and (V), respectively. In (A), (C), (E), (G), (I), (K), (M) to (O), (Q), (S), and (U), data are representative of 3 independent experiments with similar results. In (B), (D), (F), (H), (J), (L), (P), (T), and (V), quantification of the indicated protein levels was determined by Image Lab software, data are presented as mean values ± SD, and *P* values were determined by unpaired 2-tailed Student’s *t* test of *n* = 3 independent biological experiments. In (Q) and (R), data are presented as mean values ± SEM and *P* values were determined by unpaired 2-tailed Student’s *t* test of *n* = 3 independent biological experiments.

Previous studies demonstrated that ubiquitin chains added to the substrates serve as a critical signal for the cargo receptors’ recognition [39–41]. Since GSDMD could be modified by ubiquitination, we determined whether USP18 modulated the ubiquitination level of GSDMD for its degradation. We observed that USP18 could apparently promote GSDMD ubiquitination (Fig. S3A). Consistent with the data that USP18 C64A promoted the degradation of GSDMD, overexpression of USP18 C64A also led to a marked increase in GSDMD ubiquitination (Fig. 3A and B). Meanwhile, LPS alone or LPS plus ATP-induced GSDMD ubiquitination was greatly impaired in *USP18*-KD/KO THP-1-derived macrophages (Fig. S3B and Fig. 3C and D). Seven types of ubiquitination processes, including K6-, K11-, K27-, K29-, K33-, K48-, and K63-linked ubiquitination, have been reported to modulate the protein’s fate [42–44]. We then set out to define which type of GSDMD ubiquitination could be mediated by USP18 and observed that USP18 markedly increased K33-linked ubiquitination of GSDMD, but had no apparent influence on the ubiquitination of GSDMD with other ubiquitin linkages (Fig. 3E and F and Fig. S3C).

We next sought to identify which lysine (K) residues on GSDMD are associated with USP18-mediated K33-linked ubiquitination. To accomplish this, we utilized computer-assisted algorithms [45] (http://www.ubpred.org) and integrated the findings with a previous study [17]. We identified 15 potential ubiquitination sites of GSDMD and introduced arginine (R) substitutions at these sites to generate the K43R, K51R, K55R, K62R, K103R, K145R, K168R, K177R, K203R, K204R, K235R, K236R, K248R, K299R, and K387R GSDMD mutants. We found that USP18 could not further increase K33-linked ubiquitination of the GSDMD K168R mutant (Fig. S3D to G and Fig. 3G and H), and USP18 no longer promoted the degradation of the GSDMD K168R mutant (Fig. S3H and Fig. 3I and J). We next determined whether ubiquitin chains on K168 act as a degradation signal for GSDMD through a CHX chase assay, and observed that the GSDMD K168R mutant showed a slower degradation rate compared with WT GSDMD (Fig. 3K and L). Consistently, the interaction between GSDMD and p62/OPTN/NDP52 was markedly disrupted when K168 of GSDMD was mutated (Fig. 3M to P). These results suggest that K168 of GSDMD functions as a critical ubiquitination site for USP18-mediated selective autophagic degradation of GSDMD. We next sought to address the functional importance of ubiquitination of GSDMD at K168 in pyroptosis. Combined with our observation that the GSDMD K168R mutant had a longer half-life, the GSDMD K168R mutant increased CASP1/4-mediated GSDMD cleavage and pyroptosis (Fig. 3Q). To determine whether the difference in GSDMD cleavage levels between WT GSDMD and its K168R mutant was attributable to different protein levels (Fig. 3Q), we transfected the indicated plasmids into *BECN1*-KO HEK293T cells, in which macroautophagy was substantially impaired. In *BECN1*-KO HEK293T cells, we observed comparable results in GSDMD-mediated pyroptosis between WT GSDMD and GSDMD K168R-transfected cells (Fig. 3R). Additionally, the protein levels of WT GSDMD and its K168R mutant, the GSDMD-CASP1/4 interaction, and the CASP1/4-mediated GSDMD cleavage showed no difference in *BECN1*-KO HEK293T cells (Fig. 3S to V), indicating that the enhanced pyroptosis in K168R-transfected cells resulted from an elevated GSDMD protein level. Collectively, these findings suggest that USP18 promotes GSDMD degradation through ubiquitination of GSDMD at K168, thereby impairing its subsequent pyroptosis.

### E3 ubiquitin ligase MIB2 mediates the ubiquitination and degradation of GSDMD

**Fig. 4.**
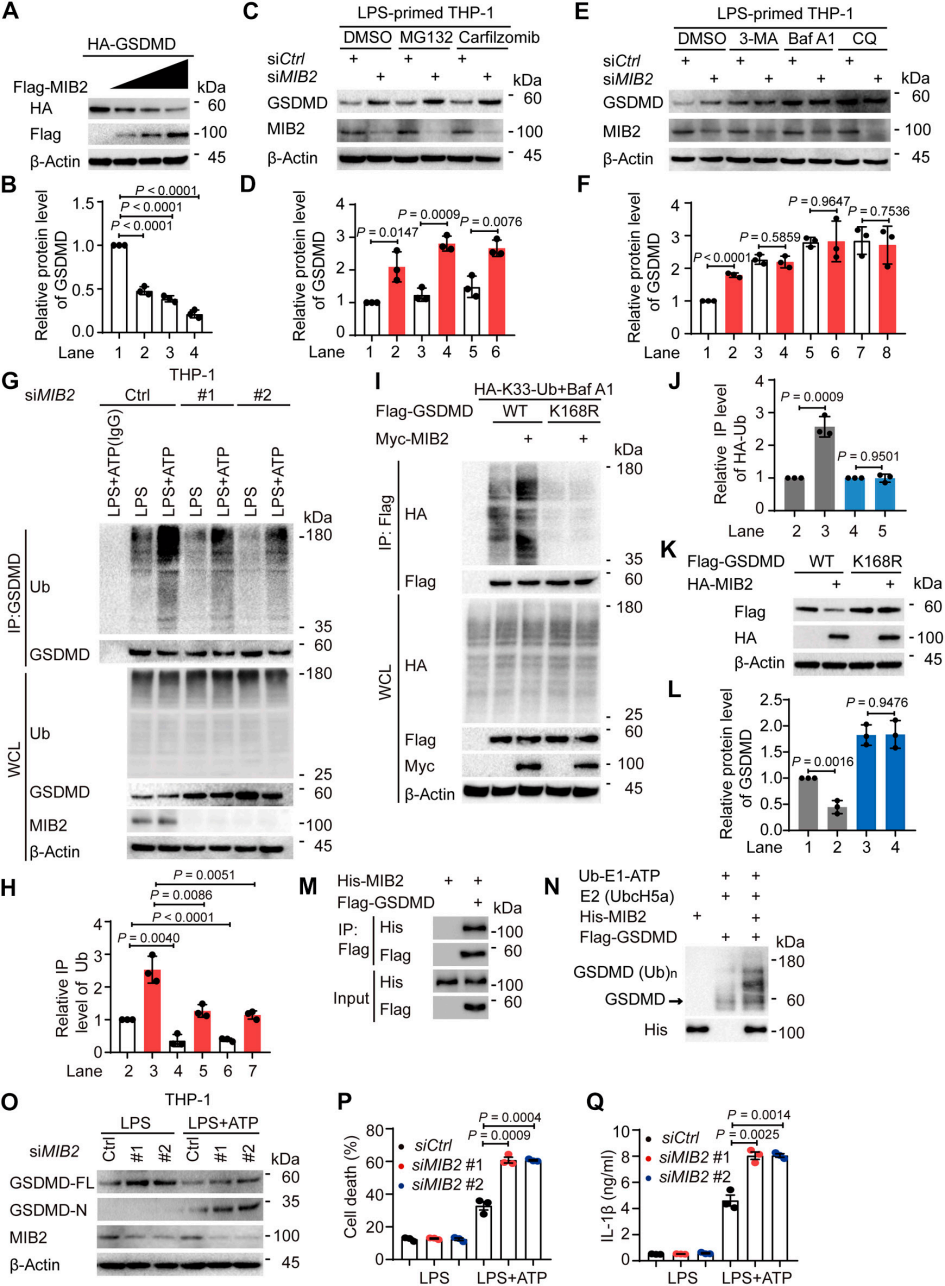
E3 ubiquitin ligase MIB2 enhances GSDMD degradation via mediating the ubiquitination of GSDMD at K168. (A and B) HEK293T cells were transfected with HA-GSDMD and increasing amounts of Flag-MIB2. Cell lysates were collected for immunoblot analysis (A). Quantification of the relative protein level of GSDMD was shown in (B). (C to F) THP-1-derived macrophages were transfected with control siRNA (si*Ctrl*) or *MIB2* siRNA (si*MIB2*) for 48 h, pre-treated with LPS (200 ng/ml) for 3 h, then treated with DMSO (vehicle), MG132 (10 μM) or carfilzomib (100 nM), 3-methyladenine (3-MA, 10 mM), bafilomycin A1 (Baf A1, 0.2 μM), or chloroquine (CQ, 50 μM) for 6 h. Cell lysates were collected for immunoblot analysis (C and E). Quantitative analysis of the relative protein level of GSDMD was shown in (D) and (F), respectively. (G and H) THP-1-derived macrophages were transfected with si*Ctrl* or si*MIB2* (#1 and #2) for 48 h, then primed with LPS (200 ng/ml) for 3 h and followed by ATP (5 mM, 3 h) treatment. Cell lysates were collected for immunoprecipitation and immunoblot analysis (G). Quantitative analysis of the relative immunoprecipitated level of ubiquitin (Ub) was shown in (H). (I and J) HEK293T cells were transfected with HA-Ub and wild type (WT) Flag-GSDMD or Flag-GSDMD K168R, together with Myc-empty vector (EV) or Myc-MIB2 for 18 h, then treated with Baf A1 (0.2 μM) for 6 h. Cell lysates were collected for immunoprecipitation and immunoblot analysis (I). Quantitative analysis of the relative immunoprecipitated level of HA-Ub was shown in (J). (K and L) Immunoblot analysis of HEK293T cells transfected with Flag-GSDMD WT or Flag-GSDMD K168R mutant, together with HA-EV or HA-MIB2 (K). Quantitative analysis of the relative protein level of GSDMD was shown in (L). (M) In vitro binding assay of 6×His-MIB2 and Flag-GSDMD proteins purified from HEK293T cells, respectively. (N) In vitro ubiquitination assay was performed in the presence of Ub, E1, E2 (UbcH5a), Flag-GSDMD, and 6×His-MIB2. (O to Q) THP-1-derived macrophages were transfected with si*Ctrl* or si*MIB2* (#1 and #2) for 48 h, then primed with LPS (200 ng/ml) for 3 h and followed by ATP (5 mM, 3 h) treatment. Cell lysates were collected for immunoblot analysis (O). Cell death (P) and production of IL-1β (Q) were assessed by LDH (lactate dehydrogenase) release assay and ELISA analysis in the supernatants, respectively. In (A), (C), (E), (G), (I), (K) and (M) to (O), data are representative of 3 independent experiments with similar results. In (B), (D), (F), (H), (J), and (L), quantification of the indicated protein levels was determined by Image Lab software, data are presented as mean values ± SD, and *P* values were determined by unpaired 2-tailed Student’s *t* test of *n* = 3 independent biological experiments. In (P) and (Q), data are presented as mean values ± SEM and *P* values were determined by unpaired 2-tailed Student’s *t* test of *n* = 3 independent biological experiments.

Since USP18 increased GSDMD ubiquitination independent of its enzymatic activity, we speculated that USP18 possibly serves as a scaffold to recruit an E3 ubiquitin ligase for ubiquitination and degradation of GSDMD. A previous study reported that E3 ubiquitin ligase SYVN1 mediated the ubiquitination of GSDMD without affecting its stability [17]. Thus, we investigated the other potential E3 ubiquitin ligases that mediate ubiquitination and subsequent degradation of GSDMD. Analysis of the UbiBrowser database revealed that MIB1/2, MDM2/4, STUB1, and RNF216 were potential E3 ubiquitin ligases involved in GSDMD ubiquitination (Fig. S4A). We transfected GSDMD with increasing amounts of indicated E3 ubiquitin ligases into HEK293T cells, and observed that only MIB2 could apparently decrease the protein level of GSDMD in a dose-dependent manner (Fig. 4A and B and Fig. S4B to F). Further assays demonstrated that *MIB2* knockdown increased the protein level of GSDMD, and this effect could be reversed by autophagy inhibitor 3-MA, Baf A1, or CQ, but not the proteasome inhibitors MG132 or carfilzomib (Fig. 4C to F). Additionally, knockdown of *MIB2* decreased LPS alone or LPS plus ATP-induced GSDMD ubiquitination (Fig. 4G and H). Consistent with the effect of USP18 on GSDMD, MIB2 failed to mediate the K33-linked ubiquitination and degradation of GSDMD K168R mutant (Fig. S4G and H and Fig. 4I to L). Additionally, we performed in vitro binding assay and in vitro ubiquitination assay, and found that recombinant His-MIB2 could directly interact with the purified Flag-GSDMD to promote the ubiquitination of GSDMD in a cell-free system (Fig. 4M and N). Given our observation that MIB2 overexpression markedly promoted GSDMD degradation, we aimed to investigate the effect of MIB2 on pyroptosis. We found that *MIB2* knockdown increased the GSDMD protein level, GSDMD cleavage level, and cell death in response to LPS plus ATP treatment in THP-1-derived macrophages (Fig. 4O and P). Consistently, *MIB2* knockdown markedly enhanced IL-1β secretion in the supernatant of THP-1-derived macrophages upon LPS plus ATP stimulation (Fig. 4Q). These results indicate that MIB2 negatively regulates GSDMD-mediated pyroptosis through enhancing ubiquitination of GSDMD at K168 and subsequent degradation of GSDMD.

### USP18 recruits MIB2 to promote the ubiquitination of GSDMD

**Fig. 5.**
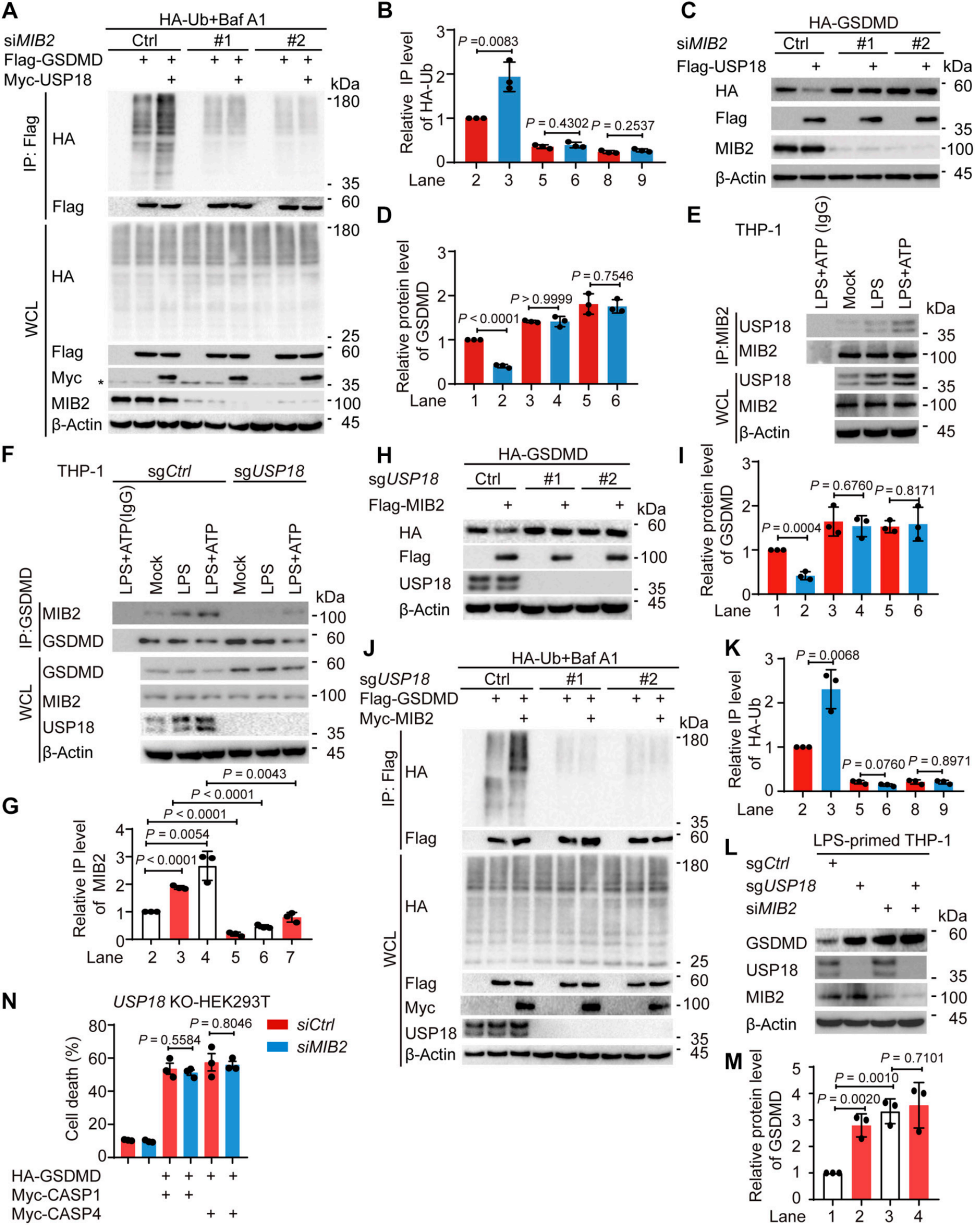
USP18 recruits MIB2 to promote ubiquitination of GSDMD. (A and B) HEK293T cells were transfected with si*Ctrl* or si*MIB2* for 36 h, then transfected with HA-ubiquitin (Ub) and Flag-GSDMD, together with Myc-empty vector (EV) or Myc-USP18 for 18 h, then treated with Baf A1 (0.2 μM) for 6 h. Cell lysates were collected for immunoprecipitation (IP) and immunoblot analysis (A). Quantitative analysis of the relative immunoprecipitated level of HA-Ub was shown in (B). (C and D) HEK293T cells were transfected with si*Ctrl* or si*MIB2* for 36 h, then transfected with HA-GSDMD and Flag-USP18 for 24 h. Cell lysates were collected for immunoblot analysis (C). Quantitative analysis of the relative protein level of GSDMD was shown in (D). (E) THP-1-derived macrophages were primed with LPS (200 ng/ml) for 3 h, followed by ATP (5 mM, 3 h) treatment. Mock, untreated. Cell lysates were collected for immunoprecipitation and immunoblot analysis. (D) THP-1-derived macrophages were transfected with si*Ctrl* or si*USP18* for 48 h, then primed with LPS (200 ng/ml) for 3 h and followed by ATP (5 mM, 3 h) treatment. Cell lysates were collected for immunoprecipitation and immunoblot analysis. (F and G) WT (sg*Ctrl*) and *USP18*-KO (sg*USP18*) THP-1-derived macrophages were primed with LPS (200 ng/ml) for 3 h, followed by treatments of ATP (5 mM, 3 h). Mock, untreated. Cell lysates were collected for immunoprecipitation and immunoblot analysis (F). Quantitative analysis of immunoprecipitated level of MIB2 was shown in (G). (H and I) WT (sg*Ctrl*) and *USP18*-KO (sg*USP18*) HEK293T cells were transfected with HA-GSDMD and Flag-MIB2 for 24 h. Cell lysates were collected for immunoblot analysis (H). Quantitative analysis of the relative protein level of GSDMD was shown in (I). (J and K) WT (sg*Ctrl*) and *USP18*-KO (sg*USP18*) HEK293T cells were transfected with HA-Ub and Flag-GSDMD, together with Myc-EV or Myc-MIB2 for 18 h, then treated with Baf A1 (0.2 μM) for 6 h. Cell lysates were collected for immunoprecipitation and immunoblot analysis (J). Quantitative analysis of the relative immunoprecipitated level of HA-Ub was shown in (K). (L and M) WT (sg*Ctrl*) and *USP18*-KO (sg*USP18*) THP-1-derived macrophages were transfected with si*Ctrl* or si*MIB2* for 48 h. Cell lysates were collected for immunoblot analysis (L). Quantitative analysis of the relative protein level of GSDMD was shown in (M). (N) *USP18*-KO (sg*USP18*) HEK293T cells were transfected with si*Ctrl* or si*MIB2* for 36 h, then transfected with HA-GSDMD and Myc-CASP1 or Myc-CASP4 for 24 h. Cell supernatants were collected for LDH release assay. In (A), (C), (E), (F), (H), (J), and (L), data are representative of 3 independent experiments with similar results. In (B), (D), (G), (I), (K), and (M), quantification of the indicated protein levels was determined by Image Lab software, data are presented as mean values ± SD, and *P* values were determined by unpaired 2-tailed Student’s *t* test of *n* = 3 independent biological experiments. In (N), data are presented as mean values ± SEM and *P* values were determined by unpaired 2-tailed Student’s *t* test of *n* = 3 independent biological experiments.

To examine the potential role of MIB2 in regulating USP18-mediated GSDMD ubiquitination and degradation, we performed siRNA-mediated knockdown of *MIB2* in HEK293T cells, and observed that *MIB2* knockdown resulted in the abrogation of USP18-mediated ubiquitination of GSDMD (Fig. 5A and B) as well as USP18-mediated GSDMD degradation (Fig. 5C and D). We next determined how USP18 regulates GSDMD ubiquitination through MIB2. Immunoprecipitation assay demonstrated that USP18 could interact with MIB2 (Fig. S5A and B and Fig. 5E). Interestingly, we observed that overexpression of USP18 as well as its enzyme inactive mutant C64A increased the GSDMD–MIB2 interaction (Fig. S5C), confirming that USP18 may function as a scaffold to bridge MIB2 to GSDMD. In addition, we found that deficiency of *USP18* impaired the GSDMD–MIB2 association (Fig. S5D to F and Fig. 5F and G). We finally investigated whether USP18 is necessary for MIB2-mediated ubiquitination and subsequent degradation of GSDMD. Overexpression of MIB2 could substantially increase the degradation of GSDMD, while *USP18* deficiency abolished MIB2-mediated GSDMD degradation (Fig. S5G and Fig. 5H and I). Notably, *USP18* deletion dramatically attenuated MIB2-mediated ubiquitination of GSDMD (Fig. S5H and Fig. 5J and K). The knockdown of *MIB2* no longer increased the GSDMD protein levels or GSDMD-mediated pyroptosis in *USP18-*deficient cells (Fig. S5I and J and Fig. 5L to N). Given the critical role of GSDMD-N in triggering pyroptosis and the location of K168 at the GSDMD-N, we sought to investigate whether USP18 and MIB2 could directly induce the degradation of GSDMD-N. We transfected the HEK293T cells with Flag-GSDMD or Flag-GSDMD-N, and found that it was challenging to detect the expression of Flag-GSDMD-N using Flag-HRP antibody (Fig. S6A), probably due to its rapid induction of cell death (Fig. S6B), which was consistent with previous studies [46,47]. Given that previous studies have demonstrated that the GSDMD-N I104N mutant is more readily detected within cells than its WT form, potentially due to decreased cell death induction (GSDMD-N I104N mutant is defective at membrane insertion) [47,48], we constructed the GSDMD-N I104N and I104N/K168R mutated plasmids and found that the expression of the indicated plasmids could be detected using Flag-HRP antibody in HEK293T cells (Fig. S6C). To investigate whether USP18 and MIB2 could directly promote the degradation of GSDMD-N, we examined whether the GSDMD I104N mutant altered the binding of USP18/MIB2 to GSDMD and whether its degradation was mediated by USP18/MIB2, ensuring the accuracy of subsequent experiments. We observed that the interaction between GSDMD and USP18/MIB2 showed no difference in WT GSDMD and I104N GSDMD-transfected cells (Fig. S6D and E). Furthermore, we found that I104N GSDMD could also be degraded by USP18 or MIB2 (Fig. S6F and G). These results suggest that it is feasible to investigate whether USP18/MIB2 can directly promote the degradation of GSDMD-N by employing the visually expressed I104N mutation. Subsequently, we observed that I104N GSDMD-N, but not I104N/K168R GSDMD-N, could be degraded by USP18 or MIB2 (Fig. S6H and I). Taken together, these results indicate that USP18 recruits MIB2 to promote the ubiquitination of GSDMD at K168 and the subsequent degradation of the full-length or N-terminal form of GSDMD, resulting in impaired pyroptosis.

### USP18 ameliorates LPS-induced inflammation in vivo

**Fig. 6.**
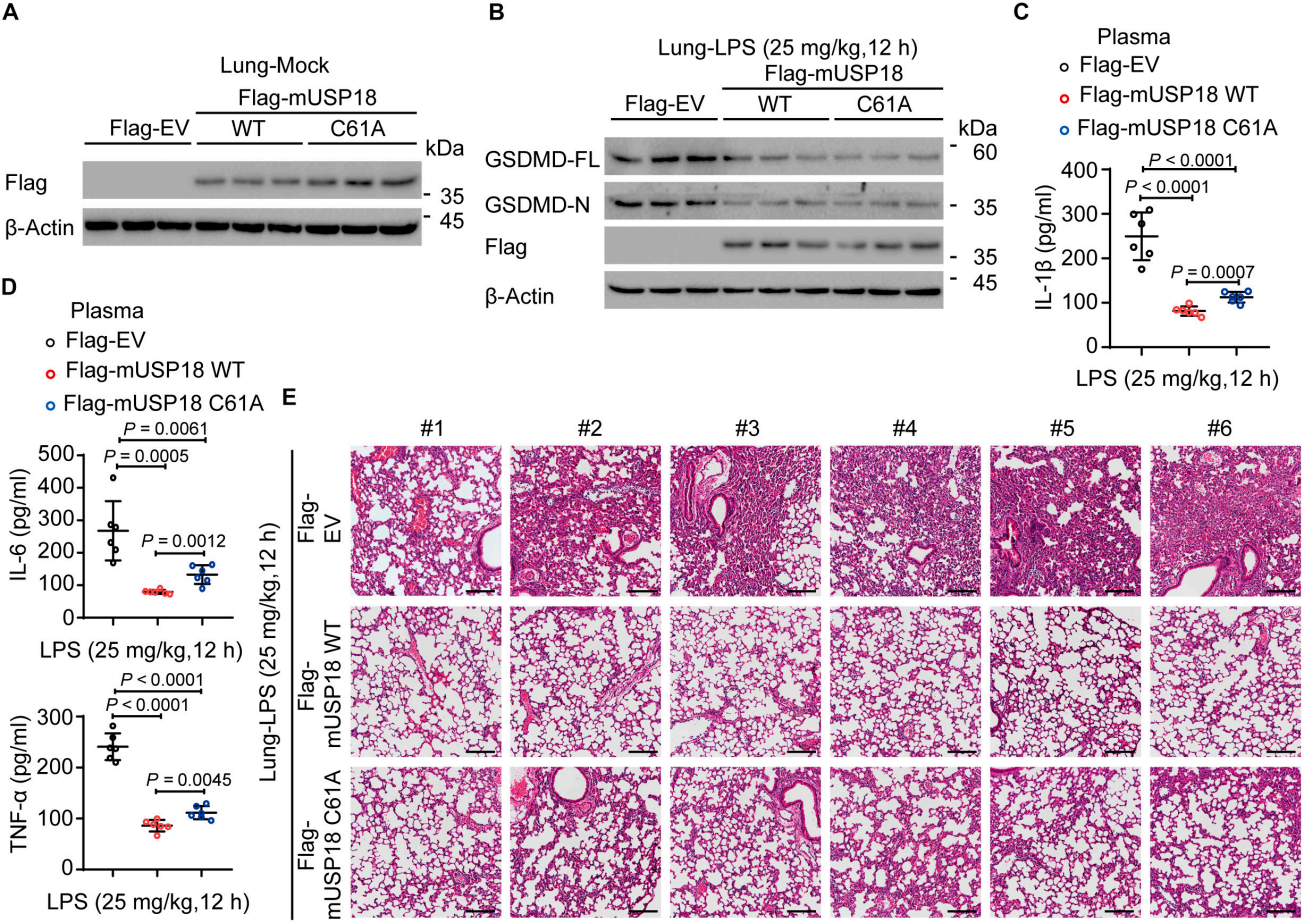
USP18 overexpression attenuates LPS-induced inflammation in vivo. (A) Immunoblot analysis of the transfected efficiency of Flag-WT mUSP18 or Flag-C61A mUSP18 plasmid in lung tissues. (B to E) Control mice or mUSP18 overexpression mice (*n* = 6 per group) after being intraperitoneally (i.p.) injected with lipopolysaccharide (LPS, 25 mg/kg mice weight, 12 h). GSDMD protein levels of the lung tissues (the mixture of 2 mice per group) were detected by immunoblot analysis (B). IL-1β, IL-6, and TNF-α release in the isolated plasma were determined by ELISA analysis (C and D). The indicated lung tissues were stained with hematoxylin and eosin (H&E) and assayed using a light microscope with ×200 magnification. Scale bars, 100 μm (E). Each symbol represents an individual mouse; data are presented as mean values ± SD and *P* values were determined by unpaired 2-tailed Student’s *t* test of *n* = 5 independent biological mice per group.

To gain further insights into the physiological role of USP18 in vivo, we established a USP18 overexpression model by administering an expression plasmid via tail vein injection, utilizing an in vivo transfection reagent. The expression of Flag-mUSP18 was confirmed in the lung tissues (Fig. 6A). We employed a model of endotoxic shock induced by *Escherichia coli* LPS and found that both USP18 WT and its enzymatic mutant C61A overexpression led to a significant reduction in GSDMD protein levels compared to control mice (Fig. 6B). Consistent with these results, we observed that overexpression of USP18 WT or its enzymatic mutation C61A resulted in the inhibition of LPS-induced secretion of IL-1β, IL-6, and TNF-α in the plasma compared to control mice (Fig. 6C and D). Although IL-1β, IL-6, and TNF-α production in the USP18 C61A-overexpressing mice was higher than that in USP18 WT-overexpressing mice (Fig. 6C and D), its enzymatic mutant C61A overexpression still led to the reduction of pro-inflammatory cytokines and a significant reduction in the lung tissue damage compared to WT mice (Fig. 6C to E). These findings suggest that USP18 could also play a crucial role in suppressing GSDMD-related inflammation in vivo through an enzymatic-dependent mechanism.

## Discussion

Pyroptosis is a form of cell death that relies on gasdermin-mediated formation pores in the plasma membrane, which subsequently promotes inflammation by releasing immunogenic cellular contents [5–7]. The inflammation-related feature of pyroptosis underscores its critical role in the host defense and homeostasis [49]. However, uncontrolled pyroptosis is strongly associated with inflammatory-related diseases, including ischemic heart disease, rheumatoid arthritis, systemic lupus erythematosus, atherosclerosis, and certain types of cancer [11,49–52]. Hence, pyroptosis must be carefully regulated to avoid unintended consequences and maintain balance. To date, although many studies highlight the importance of PTMs for gasdermin regulation, detailed mechanisms of how these PTMs modulate gasdermin-mediated pyroptosis remain to be fully investigated. GSDMD is the best-characterized member of the gasdermin family (including GSDMA-E and pejvakin) [9], and several small-molecule inhibitors of GSDMD have been developed [53–55]. However, adverse effects and low specificity limit their use. Hence, it is imperative to increase the knowledge of the mechanisms of PTM-regulated GSDMD activation, which will be a promising approach for drug development. Although ubiquitination plays a critical role in regulating protein stability, the link between ubiquitination and GSDMD stability remains to be fully elucidated during inflammasome activation. In our study, we reveal that the ubiquitination of GSDMD K168 serves as a critical signal for GSDMD degradation.

USP18, a member of deubiquitinases, has been reported to function as a “maestro” of multiple biological pathways [20], including antiviral responses and inflammation. Originally, USP18 is identified as a deISGlase, which specifically cleaves the small ubiquitin-like ISG15 from ISGlated proteins to dampen the interferon-induced overwhelmed response to viral infections [56]. Apart from its enzymatic activity, USP18 recruits USP20 to enhance an innate antiviral response through deubiquitinating STING (stimulator of interferon genes) [23]. Furthermore, USP18 acts as a scaffold protein to recruit E3 ubiquitin ligase TRIM31 for facilitating K63-linked ubiquitination and subsequent aggregation of MAVS (mitochondrial antiviral-signaling protein), which is independent of its enzymatic activity [24]. Recently, several studies also demonstrate that USP18 functions as a direct deubiquitinase to negatively regulate NF-κB signaling pathway by removing the K63-linked ubiquitin chains from TAK1 or NEMO through an enzymatic-dependent or -independent manner, respectively, resulting in inhibition of inflammation [31]. These studies indicate that USP18 is associated with distinct signaling pathways dependent or independent of its enzymatic activity. However, the functions of USP18 in GSDMD-mediated pyroptosis during inflammasome activation have not been fully discussed. Here, we uncover the novel function of USP18 in pyroptosis: during inflammasome activation, USP18 directly targets GSDMD and impairs GSDMD-mediated pyroptosis, which is independent of its enzymatic activity.

Autophagy is a highly evolutionary conserved degradation system that allows cells to rapidly eliminate various intracellular elements within eukaryotic cells [57]. The autophagy dysfunction, such as loss of the autophagy proteins, including BECN1, ATG5, or ATG16L1, contributes to excessive inflammation and hyper-activation of NLRP3 inflammasome [58–60], indicating the potential roles of autophagy in the regulation of inflammation. Autophagy, which could be induced by various inflammasome agonists, is shown to target inflammasome activators, such as intracellular DAMPs, inflammasome components, and downstream cytokines for degradation. For example, damaged mitochondria could be removed by autophagy, which leads to decreased release of mitochondria-derived DAMPs (e.g., mtRNA and mtROS) and suppression of NLRP3 inflammasome-triggered inflammation [61–63]. During the removal of inflammasome activators, autophagy is shown to be highly selective. Assembled inflammasomes undergo ubiquitination and recruit specific cargo receptor recognition, which tempers inflammation by eliminating active inflammasomes through selective autophagy [64]. Cargo receptors of selective autophagy, such as p62, NDP52, OPTN, NBR1, and TOLLIP, consist of ubiquitin-binding domains and LC3-interacting regions, which bridge the degraded substrates and autophagosomes [38,39]. Accumulating evidence has shown that ubiquitination of cargoes serves as a critical recognition signal during selective autophagy [65]. In the process of inflammasome activation, key inflammasome components, including NLRP3, ASC, and AIM2, could undergo ubiquitination, which are sensed by different cargo receptors for selective autophagy [61,64,66]. As the central pyroptosis executioner during inflammasome activation, GSDMD has been revealed to undergo ubiquitination, but the participation of cargo receptors on GSDMD stability remained to be investigated. It is interesting to discover whether GSDMD interacts with cargo receptors and can be targeted by selective autophagy to control GSDMD protein level for avoiding excessive pyroptosis and inflammation. Here, we observe that GSDMD level could be reduced by autophagy inducers such as EBSS treatment and GSDMD could undergo ubiquitination followed by multiple cargo receptors to autophagic degradation, suggesting the critical roles of selective autophagy in regulating GSDMD protein level. Previous studies demonstrated that USP18 plays a significant role in regulating autophagy: USP18 decreased paclitaxol sensitivity of triple-negative breast cancer via promoting autophagy [26]; USP18 overexpression could promote autophagy to inhibit cell apoptosis induced by spinal cord ischemia–reperfusion injury [27]; USP18 stabilizes cGAS through deubiquitination, enhancing autophagy in melanoma cells and thereby promoting resistance to vemurafenib in BRAF V600E mutant melanoma [28]. Notably, in our study, USP18 severs as a scaffold protein recruiting E3 ubiquitin ligase MIB2 to GSDMD and facilitating ubiquitination followed by multiple cargo receptor-mediated autophagic degradation of GSDMD. Overall, our study and other studies indicate that USP18 recruits distinct E3 ubiquitin ligases to mediate different ubiquitin linkage types of targeted proteins for regulating its functions, facilitating the balance of immune responses and the maintenance of homeostasis.

**Fig. 7.**
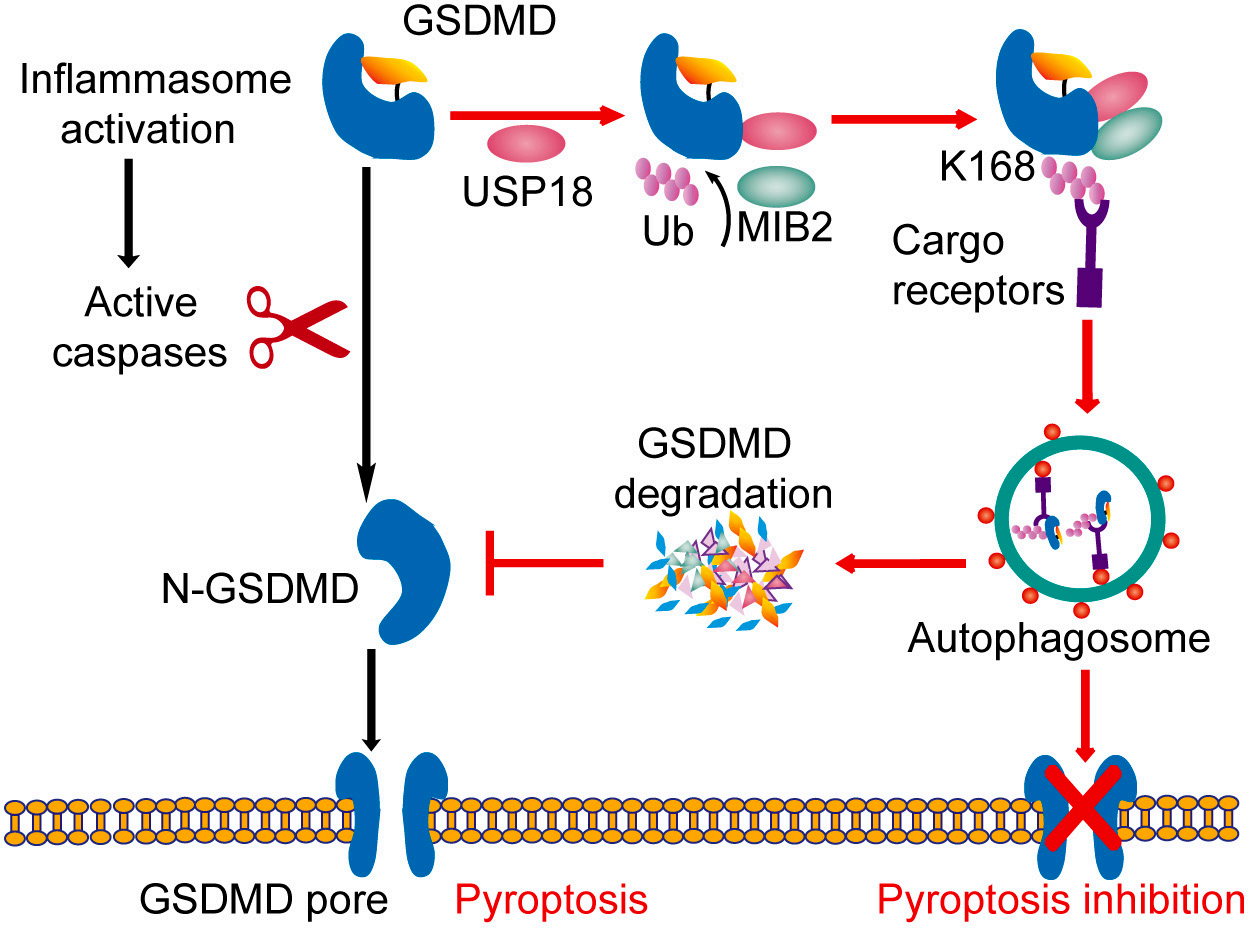
A proposed working model to illustrate how the USP18–MIB2–cargo receptors axis negatively regulates GSDMD-mediated pyroptosis. USP18 recruits MIB2 to catalyze the ubiquitination of GSDMD at K168. Through recognition of ubiquitin chains on GSDMD at K168, cargo receptors (e.g., p62, OPTN, or NDP52) deliver GSDMD to autolysosome for degradation. USP18 down-regulates the protein abundance of GSDMD, thereby inhibiting the activation of GSDMD and pyroptosis.

In summary, we identified USP18 as a negative regulator of pyroptosis by promoting the autophagic degradation of GSDMD. USP18 recruits MIB2 to catalyze the ubiquitination of GSDMD at K168. Through recognition of ubiquitin chains on GSDMD at K168, cargo receptors deliver GSDMD to autolysosome for degradation. USP18 down-regulates the protein abundance of GSDMD, thereby inhibiting the activation of GSDMD and pyroptosis (Fig. 7). Our findings link deubiquitinase (USP18), E3 ubiquitin ligase (MIB2), and autophagy to a complex regulatory network for preventing excessive GSDMD-mediated pyroptosis and uncontrolled inflammatory diseases. Hence, considering the powerful functions of USP18, pharmacological targeting of USP18 counteraction is devised to serve as a worthwhile therapeutic target for inflammation-related diseases.

## Materials and Methods

### Cell lines and culture conditions

The HEK293T cells (WT, *ATG5*-KO, or *BECN1*-KO) and THP-1 cells (monocytes and macrophages) have been described previously [61,67]. All indicated cells were grown at 37 °C in a 5% CO_2_ incubator.

### Antibodies and reagents

The antibodies specific to ubiquitin (Cat# 58395S), USP18 (Cat# 4813S), Beclin-1 (Cat# 3738), and ATG5 (Cat# 12994) were purchased from Cell Signaling Technology. Horseradish peroxidase (HRP)-anti-Flag (M2) (Cat# A8592), anti-β-actin (Cat# A1978), anti-Flag M2 affinity gel (Cat# A2220), LPS (Cat# L4524), MG132 (Cat# C2211), 3-methyladenine (3-MA, Cat# M9281), ammonium chloride (NH_4_Cl, Cat# A9434), and chloroquine (CQ, Cat# PHR1258) were purchased from Sigma-Aldrich. Bafilomycin A1 (Baf A1, Cat# S1413) was from Selleck. Anti-GSDMDC1 (Cat# sc-81868), goat anti-rabbit (Cat# sc-2004), and goat anti-mouse (Cat# sc-2005) were from Santa Cruz Biotechnology. Anti-MIB2 (Cat# A17829) was purchased from ABclonal. Anti-p62/SQSTM1 (Cat# 18420-1-AP), anti-OPTN (Cat# 10837-1-AP), and anti-CALCOCO2/NDP52 (Cat# 12229-1-AP) were purchased from Proteintech Group. Anti-hemagglutinin (HA)-HRP (Cat# 12013819001) and anti-c-Myc-HRP (Cat# 11814150001) were purchased from Roche Applied Science. Alexa Fluor 568 goat anti-rabbit IgG (H + L) (Cat# A11036) was from Invitrogen. CHX (Cat# HY-12320) and carfilzomib (Cat# HY-10455) were from MedChemExpress. Adenosine 50-triphosphate (ATP, Cat# tlrl-atpl), poly(dA:dT) (Cat# tlrl-patn-1), and flagellin (Cat# tlrl-epstfla) were from InvivoGen. Protein A agarose (Cat# 20333) and Protein G agarose (Cat# 20399) were obtained from Pierce. EDTA-free protease inhibitor (Cat# BL630B) and phosphatase inhibitor (Cat# 04906837001) were from Biosharp and Roche Applied Science, respectively. Protein marker (Cat# DB180-01) was purchased from MIKX.

### Plasmids and siRNA transfection

Plasmids of this study were cloned into the pcDNA3.1 vector (provided by Rong-Fu Wang laboratory, Houston Methodist Research Institute) for transient expression using Superluminal™ High- efficiency Transfection Reagent (MIKX, Cat# 11231804-10). The chemically synthesized siRNAs targeting specific genes were obtained from TSINGKE Biological Technology (Wuhan, China). These siRNAs were transfected into cells using Lipofectamine RNAiMAX (Invitrogen, Cat# 13778100) following the instructions provided by the manufacturer. The sequences of siRNAs used are as follows:

Control siRNA, 5′-UUCUCCGAACGUGUCACGUTT-3′

*USP18* siRNA #1, 5′-CUGCAUAUCUUCUGGUUUATT-3′

*USP18* siRNA #2, 5′-ACAUGAAGAUGGAGUGCUATT-3′

*MIB2* siRNA #1, 5′-AGAUGAUGUUGGGGUGCCGGA-3′

*MIB2* siRNA #1, 5′-UCUUGGUGUCCACCUGCUCUG-3′

*SQSTM1/p62* siRNA, 5′-CGCUCACCGUGAAGGCCUATT-3′

*OPTN* siRNA, 5′-GCACGGCAUUGUCUAAAUA-3′

*CALCOCO2*/NDP52 siRNA, 5′-GGAGGAGCUAGAAACCCUA-3′

### Generation of CRISPR/Cas9 knocked cell lines

The sgRNA sequences were designed using the CRISPR Design Tool (http://chopchop.cbu.uib.no/), synthesized by Sangon Biotech, and integrated into the leti-CRISPR v2 vector to generate the indicated gene-targeting vector. The CRISPR/Cas9 knocked cell lines were constructed according to the manufacturer’s protocols as previously described [68]. The knockout deficiency of cell lines was confirmed by immunoblot analysis. The sequences of *USP18* sgRNAs used are as follows:

*USP18* sgRNA #1: 5′-CACCGGAGTGATCACGAATGAGCA-3′

*USP18* sgRNA #2: 5′-CACCGAAGATCTGCCGGGGACTGCG-3′

### Cytotoxicity assay

The assay for cytotoxicity has been described previously [61].

### Immunoblot and immunoprecipitation analysis

The protocol for immunoblot analysis has been described previously [61,67]. For immunoprecipitation, indicated cell lysates were incubated with anti-Flag beads, anti-Myc beads, or the appropriate antibodies plus Protein A/G beads at 4°C overnight. The beads were washed 3 to 5 times with low-salt lysis buffer (LSB, 50 mM HEPES, pH 8.0, 150 mM NaCl, 1 mM EDTA, 1.5 mM MgCl_2_, 10% glycerol, and 1% Triton X-100), and the immunoprecipitates were eluted with 2× SDS loading buffer at 100 °C for 10 min for subsequent sodium dodecyl sulfate–polyacrylamide gel electrophoresis (SDS-PAGE). For the ubiquitination assay of GSDMD, 1% SDS was added to the LSB, and cell lysates were denatured at 100 °C for 5 min. Then, the denatured lysates were diluted to 0.1% SDS for immunoprecipitation with the indicated antibodies. The beads were washed several times by full immersion in LSB and eluted with 2× SDS loading buffer at 100 °C for 10 min and resolved by SDS-PAGE. Proteins were transferred to Immobilon®-PSQ Transfer Membrane (Millipore, Cat# ISEQ00010) and incubated with the appropriate antibodies. Immobilon®-Western Chemiluminescent HRP Substrate (Millipore, Cat# WBKLS0500) was used for the detection of the indicated protein. Images were obtained using the ChemiDoc MP System (Bio-Rad) and Image Lab version 6.0 (Bio-Rad Laboratories, Inc. USA).

### Recombinant proteins

The Flag-tagged GSDMD or 6×His-tagged MIB2 protein was expressed and purified from HEK293T cells, respectively. Then cells were harvested with low-salt lysis buffer supplemented with phosphatase inhibitor and EDTA-free protease inhibitor. The cell lysates were incubated with anti-Flag beads or Ni-NTA beads at 4 °C overnight. The anti-Flag beads were washed 5 times with low-salt lysis buffer, and the Flag-GSDMD protein was eluted with the low-salt lysis buffer containing 0.2 mg/ml 3×Flag-peptide (APEX Bio). The Ni-NTA beads were washed with Wash Buffer (300 mM NaCl, 50 mM NaH_2_PO_4_, and 20 mM imidazole) 5 times, and the 6×His-MIB2 protein was eluted with the Elution Buffer (300 mM NaCl, 50 mM NaH_2_PO_4_, and 300 mM imidazole).

### In vitro binding analysis

The purified 6×His-MIB2 protein was mixed with phosphate buffer saline (PBS) or purified Flag-GSDMD protein at 37 °C for 1 h and then incubated with anti-Flag beads at 4 °C overnight. The beads were washed with low-salt lysis buffer 5 times, and the immunoprecipitates were eluted with 2×SDS loading buffer at 100 °C for 10 min for subsequent SDS-PAGE.

### In vitro ubiquitination assay

The experiment was performed using an in vitro ubiquitination assay kit (abcam, Cat# ab139467) according to the manufacturer’s instructions. Briefly, purified Flag-GSDMD was incubated with ubiquitin (Ub), E1 activating enzyme, Mg-ATP, and E2 enzyme (UbcH5a, MCE, Cat# HY-P71399) with or without purified 6×His-MIB2 protein in ubiquitinoylation buffer in a total 50-μl reaction volume at 37 °C for 3 h. The assays were quenched by the addition of 50 μl of 2× non-reducing gel loading buffer, and 20 μl of each quenched assay was resolved by SDS-PAGE.

### RNA extraction and quantitative real-time polymerase chain reaction

HEK293T cells were transfected as indicated and total RNA was extracted from cells using an EZ-press RNAPurification Kit (EZBioscience, Cat# B0004D) according to the manufacturer’s protocols. Then, cDNA was generated using HiScript III RT SuperMix for qPCR (+gDNA wiper) (Vazyme, Cat# R323-01). Quantitative real-time polymerase chain reaction (qPCR) analysis was determined by using the 2× PolarSignal SYBR Green mix Taq (with Tli RNaseH) (MIKX, Cat# MKG900-10). All data were normalized to the expression of human *GAPDH*. The following primers were used:

Human *GSDMD* forward: 5′-GTGTGTCAACCTGTCTATCAAGG-3′

Human *GSDMD* reverse: 5′-CATGGCATCGTAGAAGTGGAAG-3′

Human *GAPDH* forward: 5′-GGAGCGAGATCCCTCCAAAAT-3′

Human *GAPDH* reverse: 5′-GGCTGTTGTCATACTTCTCATGG-3′

### Fluorescence microscopy

HEK293T cells (2×10^4^/ml) were cultured on glass bottom culture dishes (Nest Scientific, 801002) and transfected as indicated for 24 h. Cells were fixed with 4% paraformaldehyde for 20 min and then permeabilized in methyl alcohol for 20 min at −20 °C. After washing 3 times with PBS, cells were blocked in 6% fetal goat serum (Boster Biological, AR1009) at room temperature for 1 h and then incubated with primary antibodies diluted in 6% bull serum albumin overnight. The cells were washed 3 times with PBS, followed by a fluorescently labeled secondary antibody. Nuclei were counterstained with DAPI (Sigma-Aldrich, Cat# D9542) for 5 min. Confocal images were examined using a Leica TCS-SP8 confocal microscope (TCS-SP8, Leica) equipped with a 100× oil-immersion objective and processed for gamma adjustments using Leica AS Lite.

### Animal study

All animal experimental protocols were approved by the Animal Care Committee of Sun Yat-sen University (authorization number: SYXK (YUE) 2023-0313, Guangzhou, China). C57BL/6 WT (GDMLAC-07) mice were obtained from the Guangzhou Medical Laboratory Animal Center. Mice aged 6 to 8 weeks were selected for the experiments, as this age range is commonly used in many research studies involving mice. The mice were housed in a specific-pathogen-free (SPF) animal facility at Sun Yat-sen University. The facility maintained standard conditions, including a temperature range of 20 to 26 °C, a relative humidity between 40% and 70%, and a strict 12-h light–dark cycle (lights on at 8:00 AM and off at 8:00 PM). For in vivo transfection, Flag-mUSP18 WT (40 μg/mouse) or Flag- mUSP18 C61A (40 μg/mouse) was injected into the tail veins of mice for 48 h using an in vivo DNA transfection reagent (Entranster-in vivo; Engreen) before the endotoxic shock model was started to ensure stable expression of plasmids in mice, according to the manufacturer’s protocols and previous studies [69,70]. Then, the control mice and mUSP18 WT/C61A-overexpressing mice (6 to 8 weeks old) were intraperitoneally (i.p.) injected with LPS (25 mg/kg) for 12 h. To separate plasma, the collected blood samples were centrifuged at 500 *g* at a temperature of 4 °C for 15 min and stored at −80 °C until further analysis. Subsequently, all mice were euthanized using CO_2_ exposure, and lung tissues were collected for hematoxylin and eosin (H&E) staining and immunoblot analysis. The levels of IL-1β, TNF-α, and IL-6 in the plasma were measured using ELISA kits (Thermo Fisher Scientific, Cat# 88-7013-76; BD Biosciences, Cat# 558534; BD Biosciences, Cat# 555240), according to the manufacturer’s protocols, respectively.

### Statistical analysis

Data are presented as the mean value ± SEM or ± SD as indicated in the figure legends. All quantitative data were analyzed by unpaired 2-tailed Student’s *t* test using GraphPad Prism (GraphPad software, Inc., USA). *P* value < 0.05 was considered statistically significant.

## Data Availability

All data supporting the findings of this study are available within the article and its supplementary materials.
