# Supplementary material for: USP18 Antagonizes Pyroptosis by Facilitating Selective Autophagic Degradation of Gasdermin D
Source: Research (Wash D C). 2024 May 22;7:0380. doi: 10.34133/research.0380 (PMC11109516; doi:10.34133/research.0380)
Supplement: Supplementary 1 — Figs. S1 to S6 [file research.0380.f1.pdf]

Supplementary materials

USP18 antagonizes pyroptosis by facilitating selective autophagic degradation of GSDMD

Liqiu Wang[1,#], Mengqiu Li[1,#], Guangyu Lian[1], Shuai Yang[1], Yaoxing Wu[2], Jun Cui[1,*]

Content

Supplementary Figures 1-5

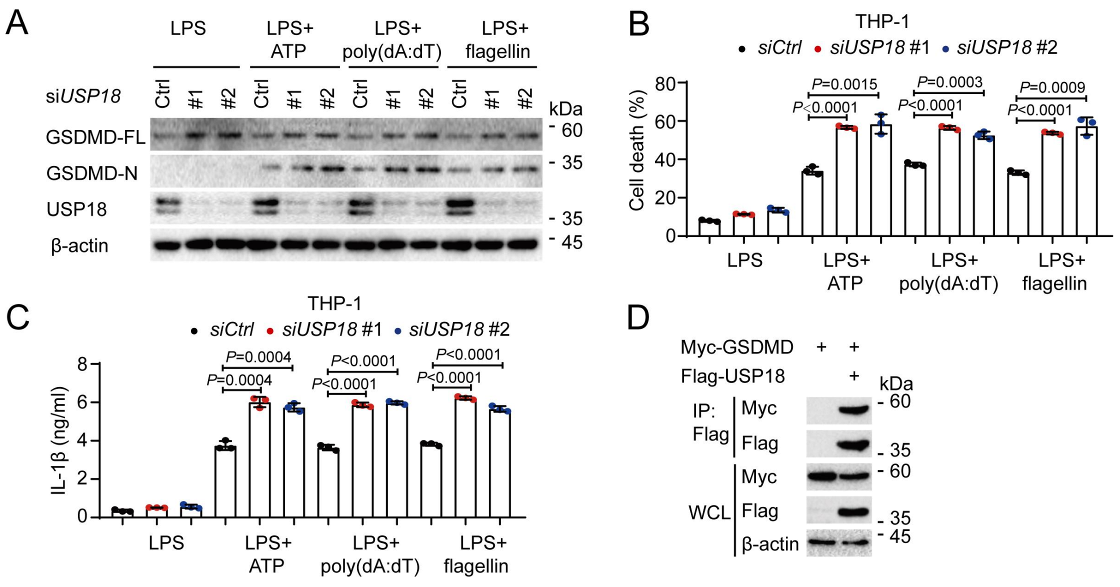

Fig. S1. *USP18* knockdown promotes pyroptosis. **(A to C)** THP-1-derived macrophages were transfected with control small interfering RNA (si*Ctrl*) or two *USP18*-specific siRNAs (si*USP18* #1, #2) for 48 h, then primed with LPS (200 ng/ml) for 3 h, followed by NLRP3 inflammasome activator ATP (5 mM, 3 h), AIM2 inflammasome activator poly (dA:dT) (2 μg/ml, 3 h) or NLRC4 inflammasome activator flagellin (1 μg/ml, 3 h) treatment. Cell lysates were collected for immunoblot analysis **(A)**. Cell death **(B)** and IL-1β **(C)** production were assessed by LDH (lactate dehydrogenase) release assay and ELISA analysis in the supernatants, respectively. **(D)** HEK293T cells were transfected with Myc-GSDMD, along with Flag-empty vector (EV) or Flag-USP18. Cell lysates were collected for immunoprecipitation (IP) and immunoblot analysis. WCL, whole cell lysates. In **(A and D)**, data are representative of three independent experiments with similar results. In **(B and C)**, data are represented as mean values ± SEM, *P* values were determined by unpaired two-tailed Student’s t test of n=3 independent biological experiments.

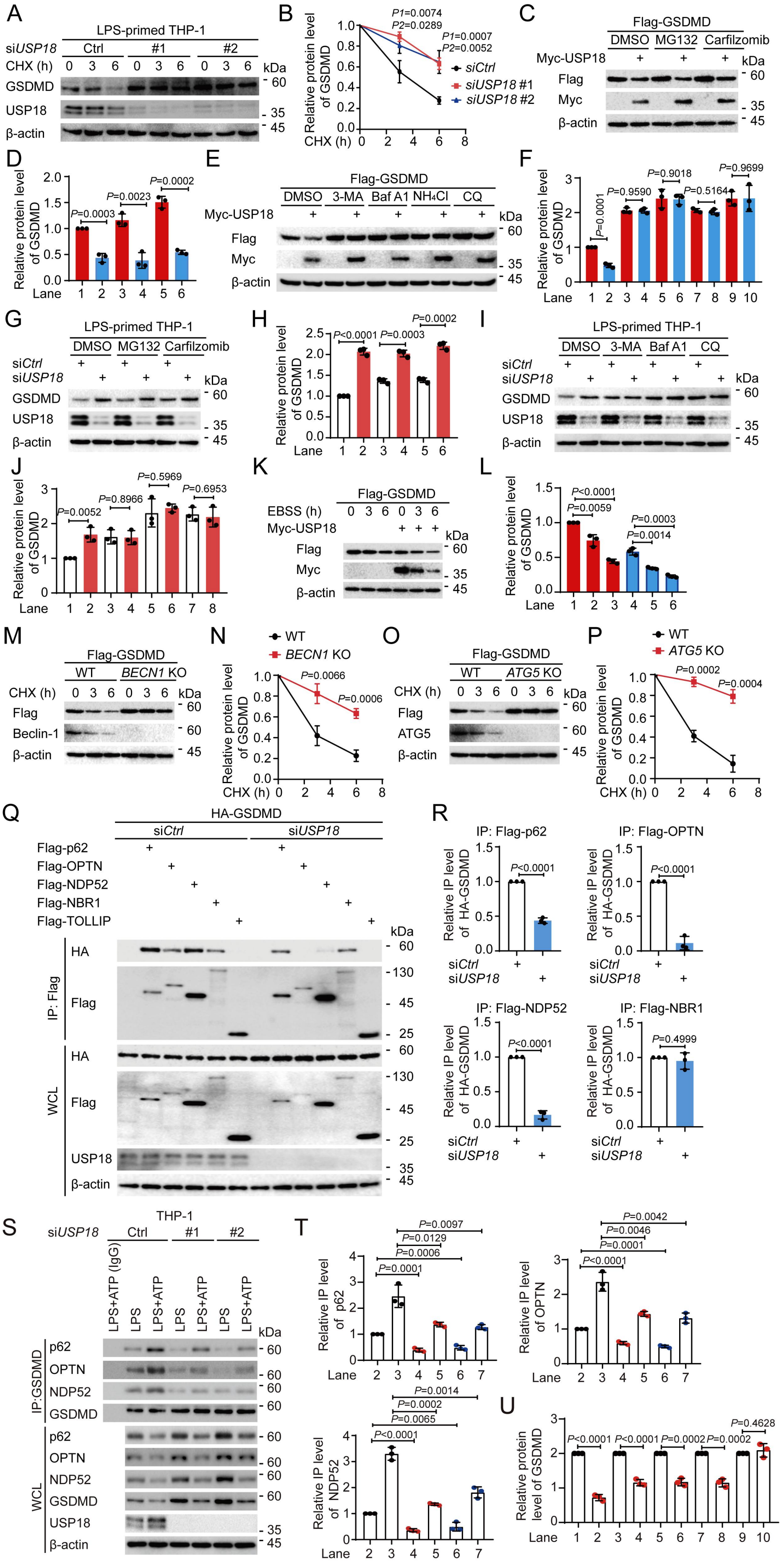

A
LPS-primed THP-1
siUSP18 Ctrl #1 #2
CHX (h) 0 3 6 0 3 6 0 3 6
GSDMD
USP18
β-actin
kDa
B
Relative protein level of GSDMD
P1=0.0074
P2=0.0289
P1=0.0007
P2=0.0052
siCtrl
siUSP18 #1
siUSP18 #2
CHX (h)
C
Flag-GSDMD
DMSO MG132 Carfilzomib
Myc-USP18
Flag
Myc
β-actin
D
Relative protein level of GSDMD
P=0.0003
P=0.0023
P=0.0002
Lane 1 2 3 4 5 6
E
Flag-GSDMD
DMSO 3-MA Baf A1 NH4Cl CQ
Myc-USP18
Flag
Myc
β-actin
F
Relative protein level of GSDMD
P=0.0001
P=0.9590
P=0.9018
P=0.5164
P=0.9699
Lane 1 2 3 4 5 6 7 8 9 10
G
LPS-primed THP-1
DMSO MG132 Carfilzomib
siCtrl
siUSP18
GSDMD
USP18
β-actin
H
Relative protein level of GSDMD
P<0.0001
P=0.0003
P=0.0002
Lane 1 2 3 4 5 6
I
LPS-primed THP-1
DMSO 3-MA Baf A1 CQ
siCtrl
siUSP18
GSDMD
USP18
β-actin
J
Relative protein level of GSDMD
P=0.0052
P=0.8966
P=0.5969
P=0.6953
Lane 1 2 3 4 5 6 7 8
K
Flag-GSDMD
EBSS (h) 0 3 6 0 3 6
Myc-USP18
Flag
Myc
β-actin
L
Relative protein level of GSDMD
P<0.0001
P=0.0059
P=0.0003
P=0.0014
Lane 1 2 3 4 5 6
M
Flag-GSDMD
WT BECN1 KO
CHX (h) 0 3 6 0 3 6
Flag
Beclin-1
β-actin
N
WT
BECN1 KO
Relative protein level of GSDMD
P=0.0066
P=0.0006
CHX (h)
O
Flag-GSDMD
WT ATG5 KO
CHX (h) 0 3 6 0 3 6
Flag
ATG5
β-actin
P
WT
ATG5 KO
Relative protein level of GSDMD
P=0.0002
P=0.0004
CHX (h)
Q
HA-GSDMD
siCtrl siUSP18
Flag-p62
Flag-OPTN
Flag-NDP52
Flag-NBR1
Flag-TOLLIP
IP: Flag
HA
Flag
WCL
HA
Flag
USP18
β-actin
R
IP: Flag-p62
IP: Flag-OPTN
IP: Flag-NDP52
IP: Flag-NBR1
Relative IP level of HA-GSDMD
P<0.0001
P<0.0001
P<0.0001
P=0.4999
siCtrl
siUSP18
S
THP-1
siUSP18 Ctrl #1 #2
LPS+ATP (IgG)
LPS
LPS+ATP
IP:GSDMD
p62
OPTN
NDP52
GSDMD
WCL
p62
OPTN
NDP52
GSDMD
USP18
β-actin
T
Relative IP level of p62
P=0.0097
P=0.0129
P=0.0006
P=0.0001
Relative IP level of OPTN
P=0.0042
P=0.0046
P=0.0001
P<0.0001
Relative IP level of NDP52
P=0.0014
P=0.0002
P=0.0065
P<0.0001
Lane 2 3 4 5 6 7
U
Relative protein level of GSDMD
P<0.0001
P<0.0001
P=0.0002
P=0.0002
P=0.4628
Lane 1 2 3 4 5 6 7 8 9 10

Fig. S2. Knockdown of *USP18* inhibits the autophagic degradation of GSDMD.

(A and B) THP-1-derived macrophages were transfected with control small interfering RNA (si*Ctrl*) or two *USP18*-specific siRNAs (si*USP18* #1, #2) for 48 h, then primed with LPS (200 ng/ml) for 3 h and followed by cycloheximide (CHX, 100 μg/ml) treatment at the indicated time points. Cell lysates were collected for immunoblot analysis **(A)**. Quantitative analysis of relative protein level of GSDMD was shown in **(B)**. **(C to F)** HEK293T cells were transfected with Flag-GSDMD, together with Myc-empty vector (EV) or Myc-USP18 for 18 h, then treated with DMSO (vehicle), MG132 (10 μM), carfilzomib (100 nM), 3-methyladenine (3-MA, 10 mM), bafilomycin A1 (Baf A1, 0.2 μM), ammonium chloride (NH_4Cl, 20 mM), or chloroquine (CQ, 50 μM) for 6 h. Cell lysates were collected for immunoblot analysis **(C and E)**. Quantitative analysis of relative protein level of GSDMD was shown in **(D and F)**, respectively. **(G to J)** THP-1-derived macrophages were transfected with control siRNA or *USP18* siRNA for 48 h, pre-treated with LPS (200 ng/ml) for 3 h, then treated with DMSO (vehicle), MG132 (10 μM) or carfilzomib (100 nM), 3-methyladenine (3-MA, 10 mM), bafilomycin A1 (Baf A1, 0.2 μM), or chloroquine (CQ, 50 μM) for 6 h. Cell lysates were collected for immunoblot analysis **(G and I)**. Quantitative analysis of relative protein level of GSDMD was shown in **(H and J)**, respectively. **(K and L)** HEK293T cells were transfected with Flag-GSDMD, together with Myc-EV or Myc-USP18 for 18 h, then treated with Earle's balanced salt solution (EBSS) as indicated time points. Cell lysates were collected for immunoblot analysis **(K)**. Quantitative analysis of relative protein level of GSDMD was shown in **(L)**. **(M to P)** Immunoblot analysis of wild type (WT), *BECN1*-knockout (KO, **M**), or *ATG5*-KO **(O)** HEK293T cells transfected with Flag-GSDMD for 18 h, then treated with CHX (100 μg/ml) as indicated time points. Quantitative analysis of relative protein level of GSDMD was shown in **(N and P)**, respectively. **(Q and R)** HEK293T cells were transfected with control siRNA or *USP18* siRNA for 36 h, then the cells were transfected with HA-GSDMD, together with Flag-empty vector (EV), Flag-p62, Flag-OPTN, Flag-NDP52, Flag-NBR1 or Flag-TOLLIP for 24 h. Cell lysates were collected for immunoprecipitation (IP) and immunoblot analysis **(Q)**. Quantitative

analysis of relative immunoprecipitated level of HA-p62, HA-OPTN, HA-NDP52, or HA-NBR1 was shown in **(R)**. **(S and T)** THP-1-derived macrophages were transfected with si*Ctrl* or si*USP18* (#1, #2) for 48 h, then primed with LPS (200 ng/ml) for 3 h and followed by ATP (5 mM, 3 h) treatment. Cell lysates were collected for immunoprecipitation (IP) and immunoblot analysis **(S)**. Quantitative analysis of relative immunoprecipitated level of p62, OPTN, or NDP52 was shown in **(T)**. **(U)** Quantitative analysis of relative protein level of GSDMD in **Fig.2Q**. In **(A**, **C**, **E**, **G**, **I**, **K**, **O**, **Q** and **S)**, data are representative of three independent experiments with similar results. In **(B**, **D**, **F**, **H**, **J**, **L**, **N**, **P**, **R**, **T** and **U)**, quantitative analysis of indicated protein levels was determined by Image Lab software, data are represented as mean values ± SD, *P* values were determined by unpaired two-tailed Student's t test of n=3 independent biological experiments.

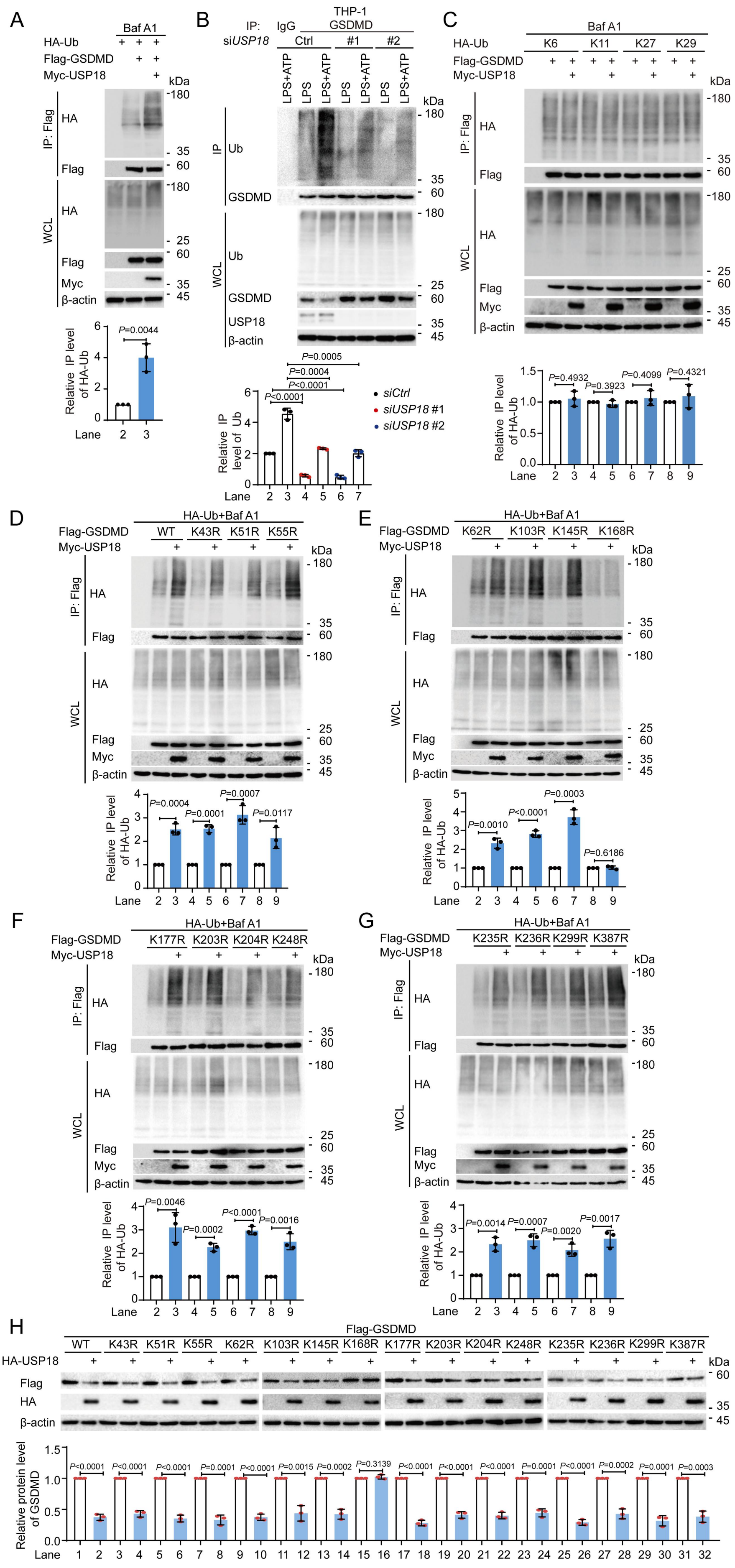
A
Baf A1
HA-Ub
Flag-GSDMD
Myc-USP18
kDa
IP: Flag
HA
Flag
WCL
Myc
β-actin
Relative IP level of HA-Ub
P=0.0044
Lane
B
IP:
siUSP18
IgG
THP-1 GSDMD
Ctrl
#1
#2
LPS+ATP
LPS
IP
Ub
GSDMD
WCL
USP18
β-actin
P=0.0005
P=0.0004
P<0.0001
Relative IP level of Ub
siCtrl
siUSP18 #1
siUSP18 #2
C
K6
K11
K27
K29
P=0.4932
P=0.3923
P=0.4099
P=0.4321
D
HA-Ub+Baf A1
WT
K43R
K51R
K55R
P=0.0004
P=0.0001
P=0.0007
P=0.0117
E
K62R
K103R
K145R
K168R
P=0.0010
P<0.0001
P=0.0003
P=0.6186
F
K177R
K203R
K204R
K248R
P=0.0046
P=0.0002
P<0.0001
P=0.0016
G
K235R
K236R
K299R
K387R
P=0.0014
P=0.0007
P=0.0020
P=0.0017
H
Flag-GSDMD
HA-USP18
Relative protein level of GSDMD
P<0.0001
P=0.0001
P=0.0015
P=0.0002
P=0.3139
P=0.0003

Fig. S3. USP18 promotes the ubiquitination of GSDMD at K168 and facilitates the subsequent degradation of GSDMD. **(A)** HEK293T cells were transfected with HA-ubiquitin (HA-Ub) and Flag-GSDMD, together with Myc-empty vector (EV) or Myc-USP18 for 18 h, then treated with Baf A1 (0.2 μM) for 6 h. Cell lysates were collected for immunoprecipitation (IP) and immunoblot analysis. WCL, whole cell lysates. **(B)** THP-1-derived macrophages were transfected with si*Ctrl* or si*USP18* (#1, #2) for 48 h, then primed with LPS (200 ng/ml) for 3 h and followed by ATP (5 mM, 3 h) treatment. Cell lysates were collected for immunoprecipitation and immunoblot analysis. **(C)** HEK293T cells were transfected with Flag-GSDMD and HA-K6-Ub, HA-K11-Ub, HA-K27-Ub, or HA-K29-Ub, together with Myc-empty vector (EV) or Myc-USP18 for 18 h, then treated with Baf A1 (0.2 μM) for 6 h. Cell lysates were collected for immunoprecipitation and immunoblot analysis. **(D to G)** HEK293T cells were transfected with HA-Ub and Flag-GSDMD WT or its mutants, together with Myc-EV or Myc-USP18 for 18 h, then treated with Baf A1 (0.2 μM) for 6 h. Cell lysates were collected for immunoprecipitation and immunoblot analysis. **(H)** Immunoblot analysis of HEK293T cells transfected with Flag-GSDMD or its mutants, together with HA-EV or HA-USP18. In **(A** to **H)**, data are representative of three independent experiments with similar results, quantification of the indicated protein levels was determined by Image Lab software, data are represented as mean values ± SD, *P* values were determined by unpaired two-tailed Student's t test of n=3 independent biological experiments.

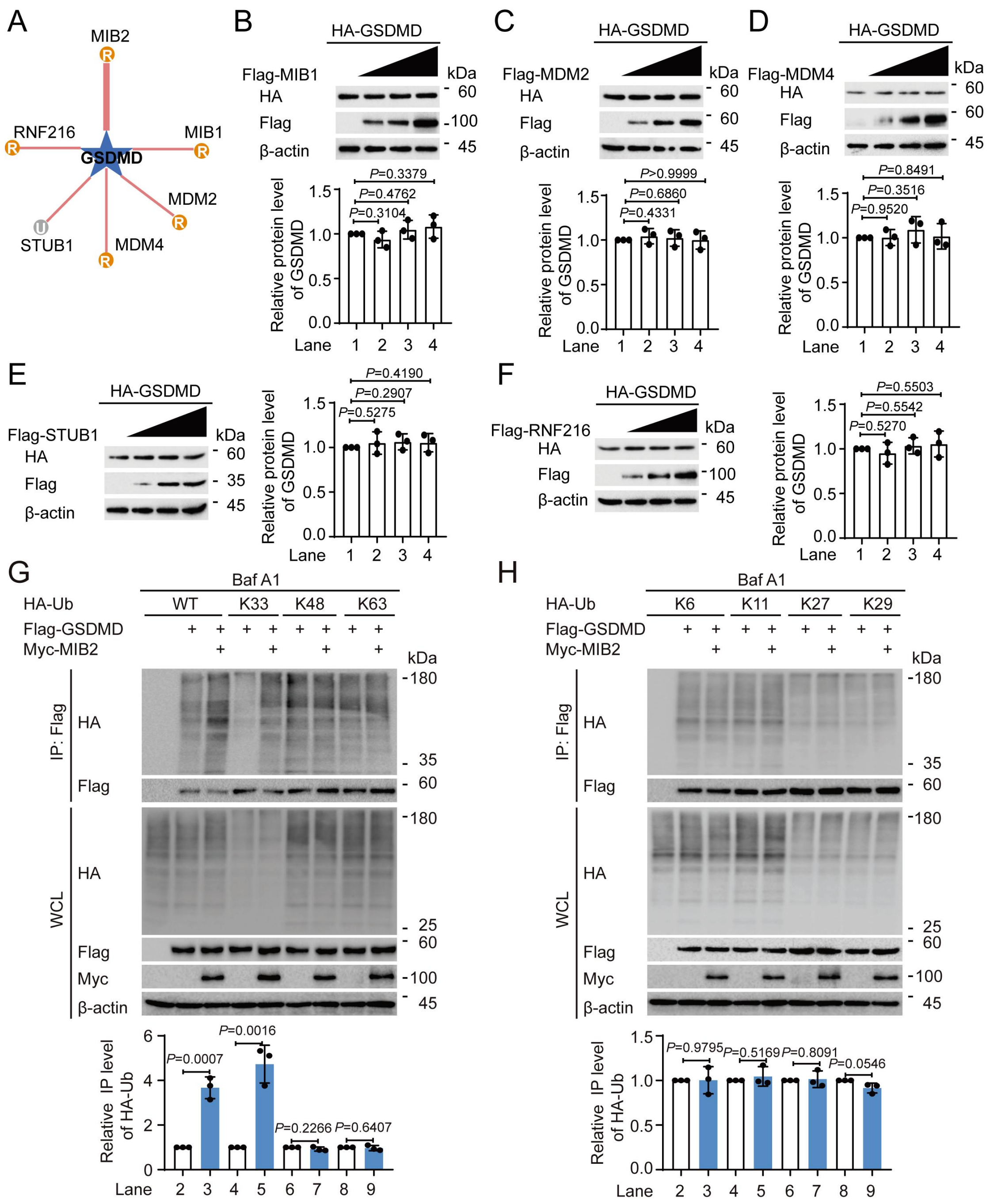

Fig. S4. Screen of E3 ubiquitin ligases that modulate the stability of GSDMD. (A) Prediction of GSDMD ubiquitination by E3 ubiquitin ligase using the Ubibrowser software. (**B to F**) Immunoblot analysis of HEK293T cells transfected with HA-GSDMD and increasing amounts of Flag-MIB1 (**B**), Flag-MDM2 (**C**), Flag-MDM4 (**D**), Flag-STUB1 (**E**), or Flag-RNF216 (**F**). **(G and H)** HEK293T cells were transfected with Flag-GSDMD and HA- wild type (WT)-ubiquitin (Ub), HA-K33-Ub, HA-K48-Ub, HA-K63-Ub, HA-K6-Ub, HA-K11-Ub, HA-K27-Ub, or HA-K29-Ub, together with Myc-empty vector (EV) or Myc-MIB2 for 18 h, then

treated with Baf A1 (0.2 μM) for 6 h. Cell lysates were collected for immunoprecipitation (IP) and immunoblot analysis. WCL, whole cell lysates. In **(B to H)**, data are representative of three independent experiments with similar results, quantification of the indicated protein levels was determined by Image Lab software, data are represented as mean values ± SD, *P* values were determined by unpaired two-tailed Student's t test of n=3 independent biological experiments.

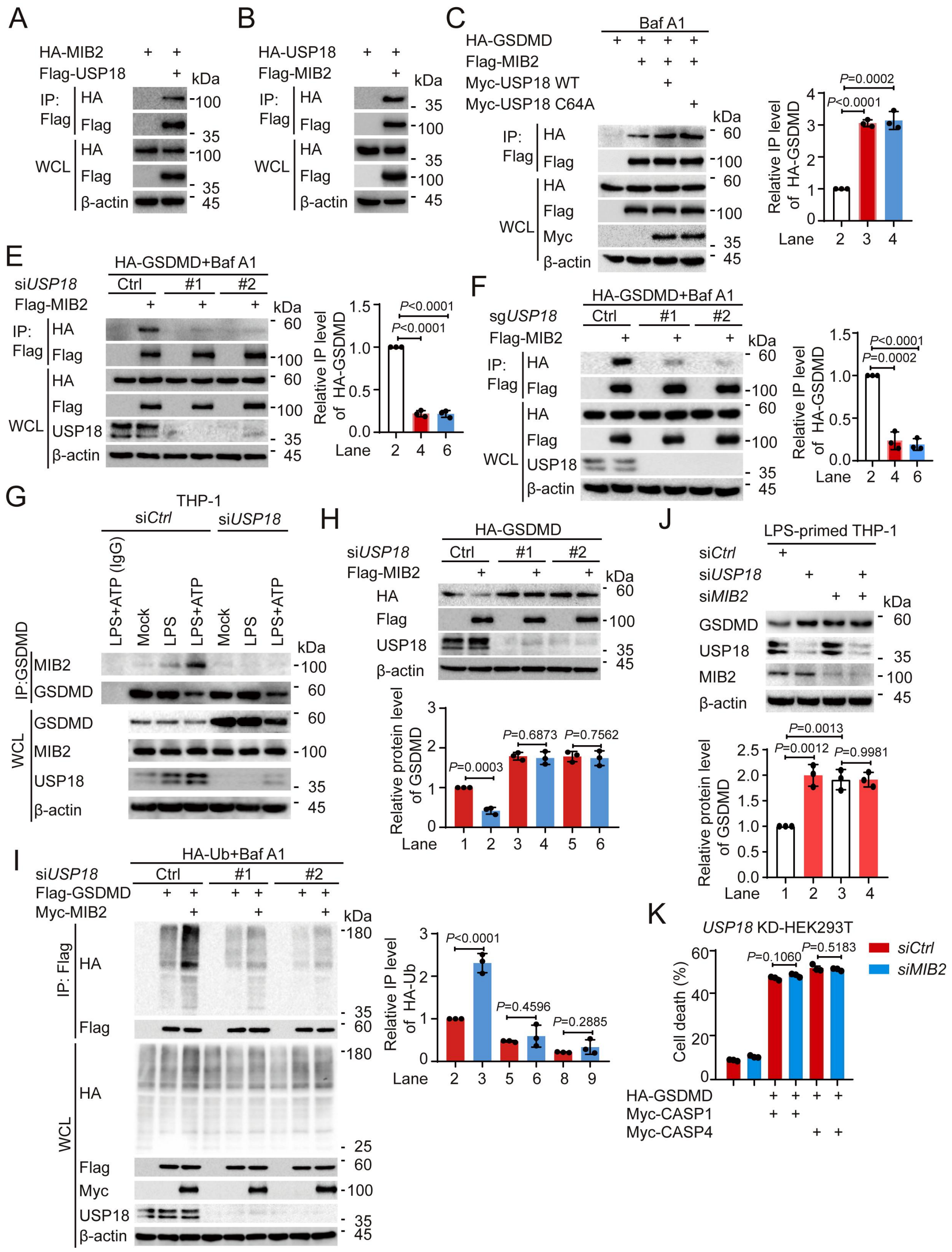

Fig. S5. USP18 recruits MIB2 to promote GSDMD degradation. (A) Immunoprecipitation (IP) and immunoblot analysis of HEK293T cells transfected with HA-MIB2 and Flag-USP18. **(B)** Immunoprecipitation and immunoblot analysis of HEK293T cells transfected with HA-USP18 and Flag-MIB2. **(C)** HEK293T cells were transfected with HA-GSDMD and Flag-MIB2, together with wild type (WT) Myc-USP18 or Myc-USP18 C64A for 18 h, then treated with Baf A1 (0.2 μM) for 6 h.

Cell lysates were collected for immunoprecipitation and immunoblot analysis. **(E)** HEK293T cells were transfected with si*Ctrl* or si*USP18* (#1, #2) for 48 h, then transfected with HA-GSDMD and Flag-MIB2 for 18 h, followed by Baf A1 (0.2 μM) treatment for 6 h. Cell lysates were collected for immunoprecipitation and immunoblot analysis. **(F)** WT (sg*Ctrl*) and *USP18*-KO (sg*USP18*) HEK293T cells were transfected with HA-GSDMD and Flag-MIB2 for 18 h, then treated with Baf A1 (0.2 μM) for 6 h. Cell lysates were collected for immunoprecipitation and immunoblot analysis. **(G)** THP-1-derived macrophages were transfected with si*Ctrl* or si*USP18* for 48 h, then primed with LPS (200 ng/ml) for 3 h and followed by ATP (5 mM, 3 h) treatment. Mock, untreated. Cell lysates were collected for immunoprecipitation and immunoblot analysis. **(H)** HEK293T cells were transfected with si*Ctrl* or si*USP18* for 36 h, then transfected with HA-GSDMD and Flag-MIB2 for 24 h. Cell lysates were collected for immunoblot analysis. **(I)** HEK293T cells were transfected with si*Ctrl* or si*USP18* for 36 h, then transfected with HA-Ub and Flag-GSDMD, together with Myc-EV or Myc-MIB2 for 18 h, then treated with Baf A1 (0.2 μM) for 6 h. Cell lysates were collected for immunoprecipitation and immunoblot analysis. **(J)** THP-1-derived macrophages were transfected with si*USP18* plus si*Ctrl* or si*USP18* plus si*MIB2* for 48 h. Cell lysates were collected for immunoblot analysis. **(K)** HEK293T cells were transfected with si*USP18* plus si*Ctrl* or si*USP18* plus si*MIB2* for 36 h, then transfected with HA-GSDMD and Myc-CASP1 or Myc-CASP4 for 24 h. Cell supernatants were collected for LDH release assay. In **(A** to **J)**, data are representative of three independent experiments with similar results. In **(C** to **J)**, quantification of the indicated protein levels was determined by Image Lab software, data are represented as mean values ± SD, P values were determined by unpaired two-tailed Student's t test of n=3 independent biological experiments. In **(K)**, data are represented as mean values ± SEM, *P* values were determined by unpaired two-tailed Student's t test of n=3 independent biological experiments.

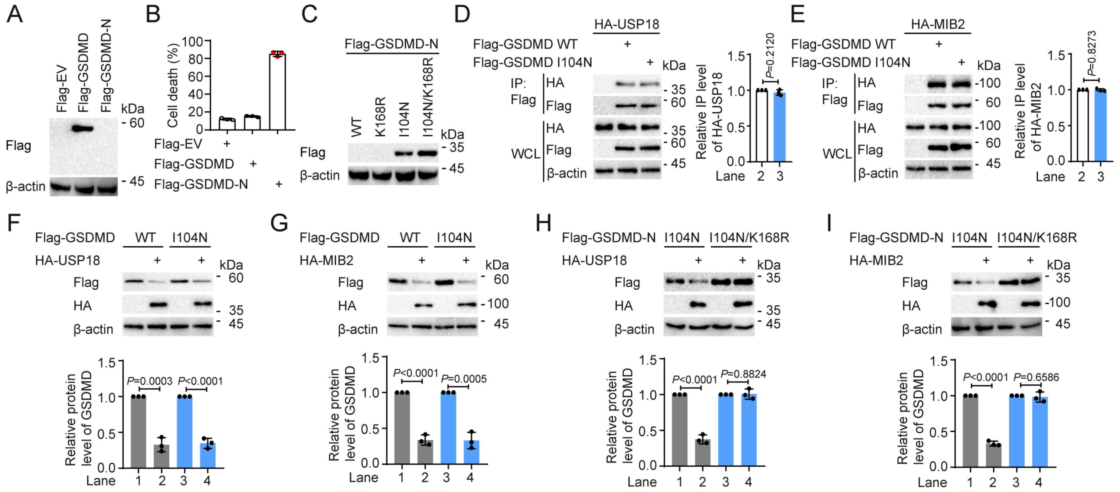

Fig. S6. USP18 and MIB2 can directly promote the degradation of N-terminal form of GSDMD. (A-B) HEK293T cells were transfected with Flag-EV, Flag-GSDMD, or Flag-GSDMD-N for 24 h, respectively. Cell lysates were collected for immunoblot analysis **(A)**, and cell supernatants were harvested for LDH (lactate dehydrogenase) assay **(B)**. **(C)** HEK293T cells were transfected with Flag-WT GSDMD-N, Flag-K168R GSDMD-N, Flag-I104N GSDMD-N, Flag-I104N/K168R GSDMD-N for 24 h, respectively. Cell lysates were collected for immunoblot analysis. **(D and E)** HEK293T cells were transfected with HA-USP18 **(D)** or HA-MIB2 **(E)**, together with Flag-EV, Flag-GSDMD, or Flag-I104N GSDMD, respectively. Cell lysates were collected for immunoprecipitation (IP) and immunoblot analysis. WCL, whole cell lysates. **(F and G)** HEK293T cells were transfected with Flag-GSDMD or Flag-I104N GSDMD, together with HA-USP18 **(F)** or HA-MIB2 **(G)**, respectively. Cell lysates were collected for immunoblot analysis. **(H and I)** HEK293T cells were transfected with Flag-I104N GSDMD-N or Flag-I104N/K168R GSDMD-N, together with HA-USP18 **(H)** or HA-MIB2 **(I)**, respectively. Cell lysates were collected for immunoblot analysis. In **(A** and **C** to **I)**, data are representative of three independent experiments with similar results. In **(D** to **I)**, quantification of the indicated protein levels was determined by Image Lab software, data are represented as mean values ± SD, P values were determined by unpaired two-tailed Student's t test of n=3 independent biological experiments. In **(B)**, data are represented as mean values ± SEM of n=3 independent biological experiments.
